# Noninvasive determination of toxic stress biomarkers by high-throughput screening of photoautotrophic cell suspension cultures with multicolor fluorescence imaging

**DOI:** 10.1186/s13007-019-0484-y

**Published:** 2019-08-24

**Authors:** Anna Segečová, María Luisa Pérez-Bueno, Matilde Barón, Jan Červený, Thomas Georg Roitsch

**Affiliations:** 1 Department of Adaptive Biotechnologies, Global Change Research Institute, CAS, 603 00 Brno, Czech Republic; 2 RECETOX, Masaryk University, 625 00 Brno, Czech Republic; 3 Department of Plant and Environmental Sciences, University of Copenhagen, 1871 Frederiksberg, Denmark; 4 Department of Biochemistry and Molecular and Cell Biology of Plants, Estación Experimental del Zaidín, CSIC, 18008 Granada, Spain

**Keywords:** Abiotic stress, Chlorophyll fluorescence, Chromium, DCMU, Fv/Fm, Glyphosate, Herbicide, Imaging, Multicolor fluorescence, Phenotyping

## Abstract

**Background:**

With increasing pollution, herbicide application and interest in plant phenotyping, sensors capturing early responses to toxic stress are demanded for screening susceptible or resistant plant varieties. Standard toxicity tests on plants are laborious, demanding in terms of space and material, and the measurement of growth-inhibition based endpoints takes relatively long time. The aim of this work was to explore the potential of photoautotrophic cell suspension cultures for high-throughput early toxicity screening based on imaging techniques. The investigation of the universal potential of fluorescence imaging methods involved testing of three toxicants with different modes of action (DCMU, glyphosate and chromium).

**Results:**

The increased pace of testing was achieved by using non-destructive imaging methods—multicolor fluorescence (MCF) and chlorophyll fluorescence (ChlF). These methods detected the negative effects of the toxicants earlier than it was reflected in plant growth inhibition (decrease in leaf area and final dry weight). Moreover, more subtle and transient effects not resulting in growth inhibition could be detected by fluorescence. The pace and sensitivity of stress detection was further enhanced by using photoautotrophic cell suspension cultures. These reacted sooner, more pronouncedly and to lower concentrations of the tested toxicants than the plants. Toxicant-specific stress signatures were observed as a combination of MCF and ChlF parameters and timing of the response. Principal component analysis was found to be useful for reduction of the collected multidimensional data sets to a few informative parameters allowing comparison of the toxicant signatures.

**Conclusions:**

Photoautotrophic cell suspension cultures have proved to be useful for rapid high-throughput screening of toxic stress and display a potential for employment as an alternative to tests on whole plants. The MCF and ChlF methods are capable of distinguishing early stress signatures of at least three different modes of action.

## Background

Plants and especially crop species are exposed to toxic stress from various sources such as pesticides, including herbicides, soil and water contaminants (use of sewage sludges, polluted areas) etc. It is important to measure toxic stress in order to study its mechanisms, select sensitive, resistant or tolerant cultivars or varieties, monitor polluted sites and help to improve plant management.

Standardized toxicity tests on plants are based mostly on vegetative endpoints such as inhibition of growth of the plant body, specific organs and structures (root, shoot, pollen tube, germination) or microscopic genotoxic assays [1–4]. The endpoints associated with biomass or growth are easily measured and have clear ecological implications. For some compounds, however, the vegetative endpoints are not necessarily the most sensitive [5] and their estimation is destructive. Also, the response requires a rather long time to manifest (days to weeks). Moreover, there is a need for an early warning system based on a multi-sensor stress-identification approach [6]. In recent years, imaging methods have gradually been used in plant phenotyping to detect and even define specific stressors such as drought, nutrient deficiency and pathogens [7, 8]. Imaging approaches allow to measure and visualize spatial as well as temporal changes in parameters based on e.g. chlorophyll fluorescence (ChlF), multicolor fluorescence (MCF), multispectral and hyperspectral reflectance and thermal imaging [9, 10]. The disadvantage of tests on plants is that they are demanding in terms of labor, space and time and the results are challenged by the heterogeneity of individual plant specimens. To overcome these drawbacks of screening tests on plants, we investigated the potential of photoautotrophic (PA) cell suspension cultures for the screening of early signals of toxic stress.

Plant cell suspension cultures represent a reduction of the complex plant body to a homogeneous mass of cells with the same identity. This makes it much easier to investigate processes at the cellular level without the interference of different cell layers and without the necessity of tissue disintegration. Cell suspension cultures are grown in axenic conditions, the cells are exposed to all treatments directly and homogeneously, which results in fast and homogeneous signal readouts. Cell suspension cultures are suitable for experiments in microtiter plates as indicated by herbicide testing [11–13], allowing for high-throughput data acquisition. Besides the application of cell suspension cultures in biotechnology [14–18] and basic research of various processes at the cellular level [19–23], successful studies of herbicides [12, 13, 24], toxicity screenings [4], salt tolerance [25] and deriving crop lines resistant to various toxicant types [26, 27] were also conducted.

Among cell suspension cultures, it is PA cell suspension cultures that are especially valuable. The presence of functional chloroplasts enables testing of substances that directly affect photosynthesis and the use of methods based on photosynthesis monitoring [13, 17, 23, 28]. PA cell suspension cultures are available for various plant species including crop, medicinal and model species [29].

This study focuses on the potential of the following selected imaging methods for detection of toxicant-specific stress response. MCF is a plant stress detection method based on excitation of the plant material with UV-radiation and detection of four characteristic peak emissions in the blue (F440), green (F520), red (F690) and far-red (F740) regions. The blue-green fluorescence is emitted by secondary metabolites in leaf tissue (mostly phenolic compounds like cinnamic acids and flavonoids) related to plant defense mechanisms [30]. Nicotinamide nucleotides, too, can contribute to the blue-green fluorescence [31]. The F440 and F520 is emitted mainly from cell walls [32]. In contrast, the F690 and F740 is emitted by chlorophyll *a*. Suitability of changes in UV-excited fluorescence or between fluorescence ratios for early stress detection was identified [30]. Despite this fact, the most typical use of MCF, especially in recent years, has been to detect plant pathogens (bacterial [33], fungal [34], viral [35], parasites [36]), drought stress [37, 38] or nutrient deficiency [39]. Only a limited number of articles discusses also successful detection of toxicity or plant susceptibility to toxic stressors (herbicides [40], heavy metals [41, 42]).

ChlF is a well-established method for indirect measurement of photosynthesis. The activity of the electron transport chain in the thylakoid responds to stress conditions [43, 44]. Indeed, numerous parameters and ratios related to photosystem II activity have been described and linked to stress [45, 46]. In toxicology, for example, ChlF is widely used with plants, algae and water macrophytes to measure effects of herbicides [46–50], heavy metals [51, 52], organic compounds [53] etc. A relevant indicator of stress is the maximum quantum efficiency of photosystem II (Fv/Fm). It is relatively easy to measure, it is widely used and its interpretation is unambiguous: healthy plants approach the theoretical maximum of 0.83 whereas decreased values signal stress [54].

Fluorescence-based methods have several advantages—they are non-invasive, the imaging approach allows for detection of early stress responses, spatial resolution of the signal heterogeneity and high-throughput data acquisition. Moreover, the amount of fluorescence parameters allows to construct signatures of specific stressors [8, 55–57] allowing for comprehensive judgment of different stress types [7]; hence their potential for bringing deeper insight into the stressors’ mode of action.

The objective of this study is to investigate the potential of PA cell suspension of *Arabidopsis thaliana* for high-throughput screening of toxic compounds with methods based on ChlF and MCF. As the cells in a suspension lack additional cell layers present, for instance, in the leaf structure, these are directly available to the tested treatments. Therefore the cell suspension could react faster than intact plants and the negative effect of the tested substances should be detected at lower concentrations [58].

## Results

In our experiments we tested the fluorescence response of *Arabidopsis thaliana* plants and PA cell suspension to several concentrations of 3-(3,4-dichlorophenyl)-1,1-dimethylurea (DCMU), glyphosate and chromium. Toxic solutions of concentrations causing distinguishable growth inhibition (Table 1d, g) were used for plants, so as to obtain a reference to an endpoint that is used in standard toxicity tests and has ecological relevance. Concentrations causing distinguishable changes in the MCF and ChlF parameters were used for the PA cell suspension. Also, the potential of some concentrations to inhibit the viability of the PA cell suspension was measured at 72 h (Table 1c). In order to compare the effective concentrations between the plants and the PA cell suspension, the concentrations of the tested solutions were recalculated per 1 g of dry weight (DW) (Table 1b, e).

**Table 1:** Concentrations of toxicants used in the experiments and comparison of the growth inhibition

Toxicant	ID	a	b	c	d	e	f	g	h	i
		PA cell suspension			Plant					
		Concentration of toxic solution	Amount of toxicant per gram DW	Viability inhibition at 72 h	Concentration of toxic solution	Amount of toxicant per gram DW	First significant decrease in projected leaf area	Growth inhibition at 21 d		
		(µM)	(mg/g DW)	(%)	(µM)	(mg/g DW)	(h)	(%)	SE	p < 0.05
DCMU	C0	0.0	0.0	0	0	0.0		0	± 5	a
	C1	0.13	0.01	NA	2	0.45	NA	5	± 8	a
	C2	0.5	0.05	3	8	1.8	120	25	± 6	a
	C3	5.0	0.52	11	16	3.6	120	68	± 7	b
	C4	50.0	5.2	14	NA					
Glyphosate	C0	0.0	0.0	0	0	0.0		0	± 8	a
	C1	100.0	7.0	− 6	50	6.0	168	56	± 7	b
	C2	250.0	19.0	NA	100	11.0	120	80	± 2	c
	C3	600.0	45.0	NA	300	34.0	72	91	± 1	d
Chromium	C0	0.0	0.0	0	0	0.0		0	± 6	a
	C1	30.0	4.0	NA	2	360.0	NA	5	± 12	ab
	C2	100.0	13.0	25	4	719.0	168	30	± 8	b
	C3	300.0	39.0	NA	8	1438.0	168	65	± 4	c
	C4	NA	NA	NA	12	2157.0	72	87	± 3	d
	C5	NA	NA	NA	20	3596.0	72	95	± 0	e

NA stands for data that were not measured (columns a–d), or not detected up to 168 h (column f). Different letters indicate significant differences (p < 0.05)

**Fig. 1:**
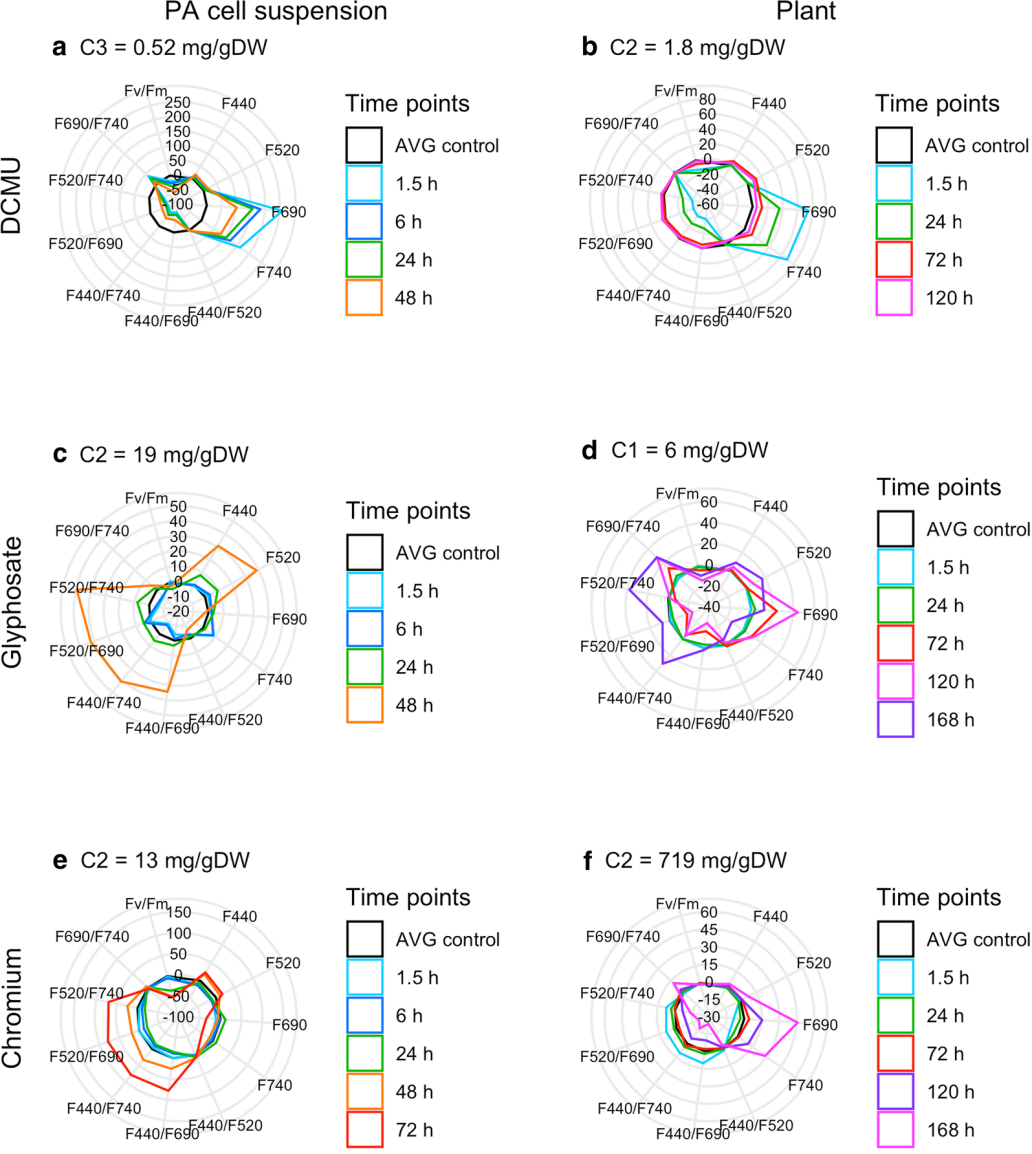
Fluorescence signatures of the tested toxicants. *Arabidopsis thaliana* suspension cultures (left column) and plants (right column) treated with DCMU, glyphosate or chromium. Evolution of stress response in time is presented for one representative concentration per toxicant

**Fig. 2:**
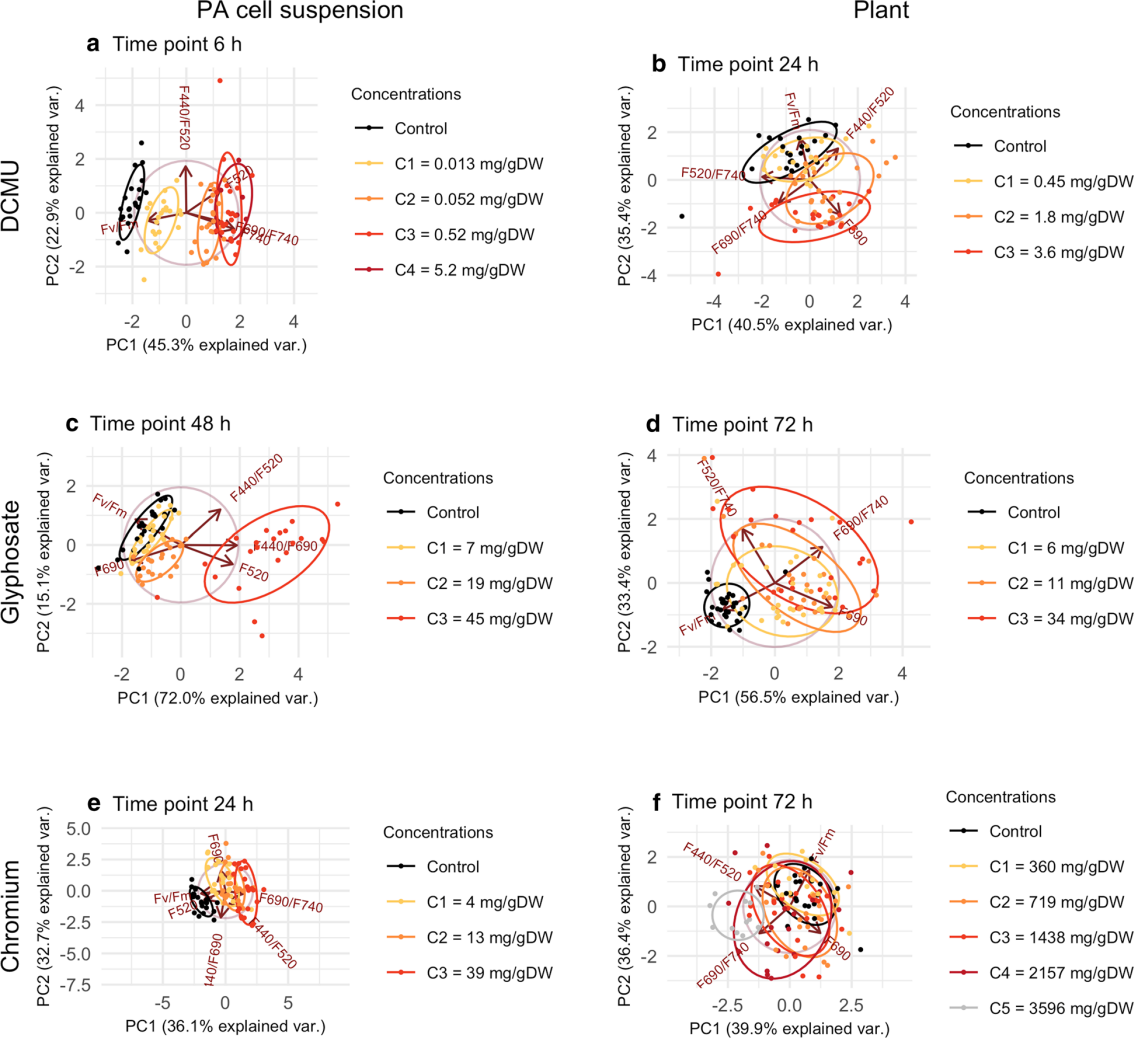
Discrimination between concentrations of the tested toxicants with PCA. *Arabidopsis thaliana* suspension cultures (left column) and plants (right column) treated with DCMU, glyphosate or chromium. The separation of groups is shown for one representative time point

**Fig. 3:**
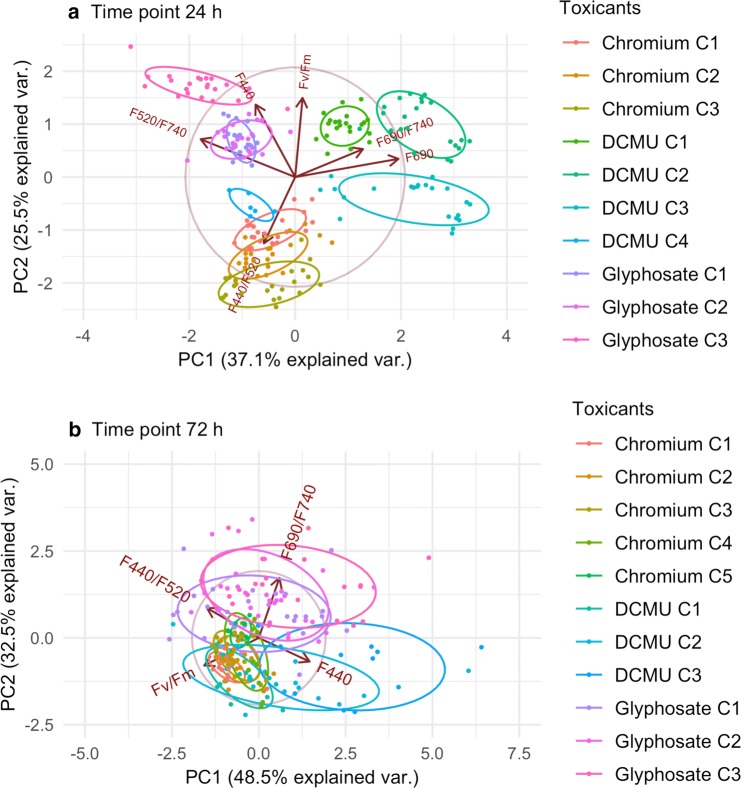
Discrimination between the toxicants with PCA. *Arabidopsis thaliana* suspension cultures at 24 h (**a**) and plants at 72 h (**b**) treated with tested concentrations of DCMU, glyphosate or chromium. One representative time point is presented for each plant material

The characteristic fluorescence signatures for each tested toxicant are presented and described for one selected concentration (Fig. 1). These are the lowest ones among the tested concentrations that still show a fluorescence response representative of the toxicant. For more information on dose-dependent characteristics of the signatures, see Additional file 1: Fig. S1–S3. For the significant differences, see Additional file 1: Fig. S4–S9. The results of PCA analyses are presented at one selected time point for each toxicant (Fig. 2–3). The selected time points show the best earliest separation of the tested groups. For more information on time-dependent characteristics of the PCA models, see Additional file 1: Fig. S10–S14. Photos showing the visual effects of the tested treatments are provided in Additional file 1: Fig. S15–S18.

### DCMU affects chlorophyll-related parameters and ratios very early

The minimum tested DCMU concentration causing detectable alterations in MCF and ChlF parameters of PA cell suspension was C1 (0.01 mg/g DW; Additional file 1: Fig. S4). Nevertheless, the results presented are those for C3 (0.52 mg/g DW), because the timeframe of the response to this concentration is more typical of a majority of the tested concentrations. The C3 caused a decrease in the viability of the PA cell suspension by 11% at 72 h (Table 1c). The response of the PA cell suspension treated with DCMU is characterized by a strong increase in F690 and F740 during the initial hours after the application of the toxicant, i.e. at 1.5 and 6 h. As demonstrated on samples treated with the C3 concentration (Fig. 1a), the values of the parameters increased from the average control values by 100 to 250%. The extent of the decrease in the ratios F440/F690, F520/F690, F440/F740 and F520/F740 (by 50 to 70%), and the increase in F690/F740 by 50% was lesser, yet still significant. All these changes tended to slightly recover at later time points, but remained significantly different from the control at all measured time points (Additional file 1: Fig. S4). Contrary to that, the decrease in Fv/Fm (by 20% at 1.5 h) became more pronounced with time. At a later time point (48 h) a slight increase in F440 and F520 (17 and 16%, respectively) was observed. F440/F520 was not indicative of any change at any measured time point. For dose-dependent character of the signature, see Additional file 1: Fig. S1a, c, e, g.

The PCA model allowed for distinctions between the DCMU-treated PA cell suspension and the control as early as at 6 h (Fig. 2a). At this time point, even the group of samples treated with the C1 concentration (0.013 mg/g DW) was separated from the cluster of groups treated with higher concentrations. All these differences were distributed in a dose-dependent manner along the first principal component (PC1) influenced mostly by F740 and Fv/Fm (for details, see Additional file 1: Table S1). At later time points, the PCA models could distinguish even between individual concentrations (Additional file 1: Fig. S10a, c, e, g).

The plants also reacted to DCMU at early time points. The minimum concentration causing detectable alterations in MCF parameters was C2 (1.8 mg/g DW; Additional file 1: Fig. S5), which caused a significant decrease in the projected leaf area at 120 h and a non-significant 25% growth inhibition at 21 d (Table 1f, g). The strongest response of the C2-treated plants to DCMU was observed at 1.5 h as an increase in values of F690 and F740 by 74 and 70%, respectively (Fig. 1b). Also the response of the ratios F440/F690, F520/F690, F440/F740, and F520/F740 was the strongest at the first time point, the values decreased by approx. 38%. The value of Fv/Fm decreased significantly, by 13%, at 1.5 h. Over time, recovery of the affected parameters and ratios was observed. Finally, at the latest measured time point (120 h), none of the responses were significantly different from the control. For dose-dependent character of the signature, see Additional file 1: Fig. S1b, d, f. For details on significant differences, see Additional file 1: Fig. S5.

The PCA model could distinguish between the treated plants and the control at 24 h, except for the group treated with the lowest DCMU concentration C1 (0.45 mg/g DW; Fig. 2b). This is in correspondence with the significant differences tested by ANOVA: no significant differences in samples treated with C1 (except for a mild and transient change in F690 at 24 h) and significant changes in most of the parameters in the samples treated with concentrations C2–3 (Additional file 1: Fig. S5).

The groups in the PCA model are distributed in a dose-dependent manner along the PC2. The between-group variance is explained mostly by Fv/Fm and F690 (for details on loadings, see Additional file 1: Table S1). The PCA models for other time points failed to bring any additional group separation (Additional file 1: Fig. S10b, d, f, h).

### The response to glyphosate is complex and gets stronger with time

For the PA cell suspension the minimum tested glyphosate concentration causing detectable changes in most of the MCF parameters was C2 (19 mg/g DW). The response of the PA cell suspension treated with glyphosate unfolds slowly in time and was detected as an increase in the values of the parameters F440, F520 and the ratios F440/F690, F440/F740, F520/F690, F520/F740 (Additional file 1: Fig. S2a, c, e, g). Significant responses of the PA cell suspension to the glyphosate concentration C2 were detected only at 48 h as changes in the above mentioned parameters by 30 to 50% (Fig. 1c; Additional file 1: Fig. S6). No significant change in Fv/Fm was observed for the C2 concentration. For dose-dependent character of the signature, see Additional file 1: Fig. S2a, c, e, g.

At 48 h, the PCA model could distinguish between groups treated with glyphosate concentrations C3 (45 mg/g DW), C2 (19 mg/g DW) and a cluster of C1 (7 mg/g DW) and the control (Fig. 2c). The variance was explained mostly by F440/F690, F520 and F690 (for details on loadings, see Additional file 1: Table S1). The PCA model for the earlier time point could discriminate only between the highest concentration C3 and the remaining groups (Additional file 1: Fig. S11a, c, e, g).

With the plants, the C1 glyphosate concentration (6 mg/g DW) caused a significant decrease in the projected leaf area at 168 h and inhibited the plants’ growth by 56% at 21 d (Table 1f, g; for time-dependent trend, see Additional file 1: Fig. S7l). The response of the plants to glyphosate as illustrated on the C1 concentration is characterized by a significant decrease in Fv/Fm (by 1%) at 24 h, followed by an increase in F690 (24%) and F690/F740 (13%) and a decrease in F440/F690 (16%) and F520/F690 (18%) at 72 h (Fig. 1d). These responses got stronger at 120 h. At 168 h, the trend of the signature changed and was characterized by recovery of the previously sensitive parameters and significant changes in F740 (decrease by 14%) and an increase in the related ratios F440/F740, F520/F740 and F690/F740 (30, 38 and 29%, respectively). For significant differences, see Additional file 1: Fig. S7. For dose-dependent character of the signature, see Additional file 1: Fig. S2b, d, f.

At 72 h, our PCA model for the plants could discriminate between the cluster of samples treated with higher concentrations C2–C3 (11–34 mg/g DW) and the control (Fig. 2d). It was not possible to identify individuals treated with the lowest tested concentration C1 (6 mg/g DW) from either the control or the higher concentrations. There is an apparent dose-dependent trend in the distribution of the groups in line with the opposing Fv/Fm and F690/F740 (for details on loadings, see Additional file 1: Table S1). The PCA models for other time points were unable to bring additional group separation (Additional file 1: Fig. S11b, d, f, h, i).

### The response to chromium is complex and gets stronger with time

With the PA cell suspension, the C2 concentration of chromium (13 mg/g DW) caused 25% inhibition of viability at 72 h (Table 1c). The reaction of the PA cell suspension to chromium grew stronger at the later time points (Fig. 1e). The first significant responses were detected at 1.5 h as a decrease in F690 (10%) and at 6 h as a decrease in F520 (13%) and Fv/Fm (5%) (Additional file 1: Fig. S8). The strongest response was observed at 72 h as pronounced changes that had already been observed at 48 h. The response is characterized by a strong increase in the values of the ratios of F440/F690, F440/F740, F520/F690, F520/F740 (70 to 80%), a moderate increase in F440 and F520 (23 and 17%, respectively) and a decrease in the values of F690, F740 and Fv/Fm (34, 32 and 53%, respectively). For dose-dependent character of the signature, see Additional file 1: Fig. S3a, c, e.

Our PCA model for the PA cell suspension clearly distinguished all chromium-treated groups from the control group at 24 h. The arrangement of the groups follows the dose-dependent trend in the directions of Fv/Fm and F520; the model, however, was not able to distinguish between the tested concentrations (for details on loadings, see Additional file 1: Table S1; for changes in PCA models for different time points, see Additional file 1: Fig. S12a, c, e, g, i).

Regarding the plants, the first negative impact of the C2 chromium concentration (719 mg/g DW) on their growth was detected at 168 h as a decrease in the projected leaf area and at 21 d as a 30% growth inhibition (Table 1f, g; for time-dependent trend, see Additional file 1: Fig. S9l). The early response of the plants to chromium at 1.5 h is characterized by a moderate rise in the ratios of F440/F690, F440/F740, F520/F690, F520/F740 (8 to 11%) and a decrease in F690 and F740 (both 9%; Fig. 1f; for significant differences, see Additional file 1: Fig. S9). Most of these changes were followed by a reversion to the control values at 72 h. At later time points, the response shifted to the opposite trends with the strongest changes at 168 h: a strong increase in F690 and F740 (46 and 28%, respectively), a moderate increase in F690/F740 (12%) and a decrease in the ratios of F440/F690, F440/F740, F520/F690, F520/F740 (13 to 24%) and Fv/Fm (1%). For dose-dependent character of the signature, see Additional file 1: Fig. S3b, d, f, g, h.

The PCA model of the chromium-treated plants at 72 h could not discriminate between the control and the treated groups but for one exception: separation of the group treated with the highest concentration C5 (3596 mg/g DW) from the cluster of control and C1–C2 (360–719 mg/g DW) (Fig. 2f). The result is comparably influenced by PC1 and PC2 (for details on loadings, see Additional file 1: Table S1). The PCA models for earlier or later time points provided no better distinction between the groups (Additional file 1: Fig. S12b, d, f, h, j).

### Specific responses to toxicants can be identified on PA suspensions better than on plants

The PCA model for the PA cell suspension fed with pooled data for all the tested toxicants measured at 24 h clearly separated the groups treated with different toxicants from each other to opposite parts of the plot (Fig. 3a). The only exception concerns the group treated with the highest DCMU concentration (C4, 5.2 mg/g DW). The PC1 separates the C1, C2 and C3 (0.013–0.52 mg/g DW) DCMU-treated groups from each other and from the rest of the toxicants. This is mostly influenced by F690 and F520/F740. The PC2 separates C4 DCMU along with all the chromium-treated groups from the glyphosate- and DCMU-treated groups. Moreover, PC2 also describes dose-dependent differences within individual toxicants. PC2 is mostly influenced by Fv/Fm, F440, and F440/520 (for details on loadings, see Additional file 1: Table S1). For changes in PCA models at other measured time points, see Additional file 1: Fig. S13.

The PCA model for the plants fed with all the tested toxicants measured at 72 h distributes the groups in clusters according to the toxicants (Fig. 3b). Most of the groups do overlap, yet there is an apparent separation of the glyphosate-treated and the DCMU-treated groups along the PC2. This separation is mostly influenced by F690/F740 (for details on loadings, see Additional file 1: Table S1).

The model did not differentiate between individual concentrations of the same toxicant, nor could it distinguish the chromium-treated groups from the other two toxicants. For changes in PCA models at other measured time points, see Additional file 1: Fig. S14.

## Discussion

The aim of this study was to test the potential of PA cell suspension cultures for high throughput screening of toxic compounds by fluorescence imaging methods in order to generate relevant information on toxicity in plants and to reduce the extent of laborious plant tests for the purpose of screening or cellular-level investigation. In general, the PA cell suspension reacted to all the tested stressors at earlier time points and with higher amplitude of the signal compared to the plants. Also, the PA cell suspension was more sensitive to lower concentrations than the plants. Such quantitative differences in the response of the PA cell suspensions compared to the whole plants can be attributed to different degrees of exposure to the toxicant due to several factors including the absence of a cuticle and cell layers, somaclonal variation and leakage of the toxicants from the cells into the nutrient medium [24].

We were able to detect fluorescence responses to all tested toxicants in the entire tested concentration range in both the PA cell suspension and the whole plants. All negative effects of the tested toxicants exhibited as decrease in the projected leaf area of plants were also detected with fluorescence parameters at earlier time points. In some cases, transient stress signals that recovered over time but still resulted in inhibited growth at later time points (e.g. DCMU-treated plants) were also detected by changes in MCF and Fv/Fm. Moreover, mild and transient stress not having a significant effect on the final plant DW could also be detected (plants treated with low doses of DCMU or chromium). Such subtle stress could play a significant role if the plant was to be exposed to more stress factors—as is usually the case in more natural, non-lab conditions. This highlights the potential of MCF and ChlF parameters for early stress screening applications.

The responses to toxicants with different modes of action differed. It would not be trivial to describe individual characteristic signatures because the responses underwent dynamic changes over time. In some cases, complete changes in trends were observed for particular parameters. For illustration of this phenomenon, compare the responses of F740 in the plants: DCMU caused an early response, the signal rises; glyphosate caused a late response, the signal decreases; chromium caused an early decrease and a later increase of the F740 signal. Nevertheless, the differences in the characteristic signatures can be conveniently compared via visualization in spider graphs covering also the temporal dynamics. To compare the responses to all the tested compounds and their concentrations within one graph, the principal component analysis (PCA) was used. PCA was also used to investigate the potential of MCF and ChlF to discriminate between toxicants with different modes of action. This method is very efficient in dealing with multidimensional data sets. It can serve for selecting the most informative variables [59]. Moreover, PCA reduces the number of dimensions of the data set into fewer artificial variables—i.e. principal components—based on the combination of the selected original variables, while still maintaining most of the variability of the data set [60]. This allows to visualize the relationships and variability of the observations in a two-dimensional plot called a biplot. Patterns and relationships among the observations can be identified, making the multidimensional data diagnosis and interpretation much easier.

Biplots can serve for deeper investigation of how the informative parameters define a given data set, identify relations between these parameters (clustering of parameters with a similar effect on the data, the length and angle of the arrow showing the power of the parameter for each principal component) and their influence on the distribution of observations among them (clustering of observations with a common response in the selected parameters), greatly facilitating multidimensional data diagnosis and interpretation.

### DCMU

Responses of both the PA cell suspension and the plants were the strongest at the earliest measured time point and the magnitude of the response weakened with time. As expected, the response was characterized by changes in parameters and ratios related to chlorophyll (F690, F740 and ratios including these; Fv/Fm) [61]. The F690/F740 was not affected significantly in the plants. The increase in F440 and F520 observed with the PA cell suspension at later time points suggests changes in stress-related secondary metabolism (e.g. changes in pigment composition and flavonoid production) [30] or changes in the concentration of nicotinamide nucleotides [31].

The PA cell suspension was more sensitive, showing stronger response even to lower concentrations than the whole plants. Yoneyama et al. [62] suggested that the reason behind differences in sensitivity of PA cell suspensions and plants is the difference in the permeability of DCMU into the cell walls and membranes.

Interestingly, the plant response to DCMU observed at 1.5 h as changes in F690, F740 and all related ratios correlated with the decrease in the projected leaf area at 120 h in a dose-dependent manner. After proper validation, this correlation could be used for prediction of growth inhibition during DCMU treatment of plants.

The PCA model of the PA cell suspension treated with DCMU was much more successful in distinguishing individual tested concentrations and even at earlier time-points compared to the model of the DCMU-treated plants. Nevertheless, the fluorescence signature and PCA model of plant response to DCMU detected transient stress response to concentrations with no significant final growth inhibition. This demonstrates the potential of MCF and ChlF signatures to detect even mild or transient stress that does not necessarily result in ultimate growth inhibition, but could significantly add to the overall negative stress effect in case of multiple stressors.

### Glyphosate

Contrary to the responses to DCMU, the response of the PA cell suspension as well as the plants treated with glyphosate was increasing with time. The response of the PA cell suspension was characterized by an increase in F440, F520, their ratios over F690 and F740 and no effect on Fv/Fm (a significant decrease of Fv/Fm was caused only by the highest tested concentration). In contrast, the response of the plants was characterized by changes in their chlorophyll related parameters: Fv/Fm, F690, F740 and corresponding ratios, even though glyphosate does not primarily inhibit photosynthesis [63]. The early negative effect of glyphosate on the ChlF induction and Fv/Fm of *Arabidopsis* was also observed by Barbagallo et al. [43]. It has been explained by disruption of metabolic reactions not involved in photosynthesis but affecting pool sizes of metabolic intermediates, having potential feedback on photosynthetic processes. Glyphosate is known to concentrate in meristematic tissues and inhibit source-sink processes, such as a decrease in photosynthesis and in sucrose synthesis and translocation [64], being a probable cause of the differences in the signatures of individual cells in PA cell suspensions and plants with complex source-sink relations.

Regarding the rapidity of plant stress detection at early time points, the Fv/Fm and MCF parameters are sensitive to glyphosate doses causing 50 to 100% final growth inhibition at 21 d and can reveal negative changes before any effect on the rosette size becomes apparent.

The PCA models visualizing the PA cell suspension and the plants treated with glyphosate proved that PA cell suspensions are more sensitive to glyphosate than whole plants.

### Chromium

The responses of the PA cell suspension and the plants to chromium also increased over time. Chromium is known to cause a decrease in Fv/Fm across the plant kingdom in monocots [65], dicots [66], mosses [67] and unicellular algae [68]. In our experiments, the decrease in Fv/Fm was significant for both of the plant materials but the PA cell suspension reacted much more strongly and faster than the plants. Both the PA cell suspension and the plant samples reacted with an immediate and transient decrease in the chlorophyll-related parameters F690, F740 and their ratios with F440 and F520. Judging from the nature of the response, it might be attributed to an osmotic shock caused by application of chromium into the medium, as heavy metals are known to cause extrusion of potassium and hydrogen cations from the root cells [69].

At the later time points, the response of the PA cell suspension was characterized by an increase in F440 and F520 and a decrease in F690 and F740, while the plants reacted with a strong increase in F690 and F740 and only a slight increase in F440 and F520. Both the PA cell suspension and the plants reacted to chromium with changes in the ratios of F440 or F520 to F690 or F740. This is in correspondence with results from plants treated with heavy-metal mixtures [41].

Overall, the PA cell suspension reacted to chromium concentrations two orders of magnitude lower and at earlier time points than the plants. The signature of chromium treatment found for the PA cell suspension is similar to that found for the plants at early time points, and at later-time-point response of plants treated only with the highest chromium concentration. All of the above mentioned differences could probably be attributed to differences in chromium application. The PA cells in the suspension were exposed to the chromium solution directly. The plants, however, were exposed via roots, where chromium had to be taken up, translocated into the shoot and into the photosynthetically active mesophyll cells. Moreover, the translocation of chromium from the root to the shoot is low [70], hence the higher concentration and longer exposition time needed to obtain significant fluorescence responses from plants. Nevertheless, there is evidence that translocated chromium ions can cause significant damage or alteration in morphology (guard cell shape and stomatal pore size) or processes in leaf cells, including damage to chloroplasts and mitochondria via ROS formation and competition for nutrient uptake [71].

The above discussed results show that the responses of MCF and ChlF measured parameters were specific to toxicants with different modes of action and that the development of the response changes in time.

The F690/F740 ratio has been described as a stress marker. Buschmann and Lichtenthaler [30] reported specific changes related to short- and long-term stress. However, in our case, this parameter was not equally sensitive to all toxicants and plant materials. Particular indices could be used for rapid screening purposes rather than counting on markers of general stress, since they could be relatively insensitive at early time points. Moreover, using methods based solely on photosynthesis monitoring can fail to identify non-photosynthesis targeted stressors [72, 73]. Therefore, in order to develop stressor-specific signatures, it is recommended to combine different signals (in our case MCF and ChlF parameters) and also to take into account temporal characteristics of the stressor, as was also pointed out by Chaerle et al. [6].

It was observed that following groups of parameters and ratios displayed a high degree of correlation: (a) group of F440, F520; (b) group of F680, F740; (c) group of the ratios F440/F690, F440/F740, F520/F690, F520/F740; it was therefore possible to select just one representative of each group to be used in the PCA model. Consequently, parameters from the same group can be treated almost interchangeably for the purposes of the PCA models and the models should therefore be compared from the perspective of groups of parameters rather than from the perspective of individual parameters.

### Distinguishing between the toxicants

In order to investigate the potential of the tested MCF and ChlF parameters and ratios to distinguish between toxicants with different modes of action, PCA was used to visualize and explore the characteristic signatures for each toxicant in a comprehensive manner and in a format allowing easy comparison.

The results obtained from experiments with the PA cell suspension showed that the PCA model for pooled data for all three tested toxicants could very comprehensively visualize relationships between their individual concentrations and also explain the variation between the groups treated with different toxicants. Very good visual separation of the treated groups was obtained already at 24 h. The groups fell into separate sectors of the biplot based on the treatment with each particular toxicant. This highlights the differences in the mode of action of the tested toxicants. Moreover, the placement of the group treated with the highest DCMU concentration close to the chromium-treated cluster suggests some similarities in the response of the PA cell suspension to high DCMU and low chromium treatment. Physiological interpretation would require further investigation; nevertheless, the potential of PCA for finding interesting relations or clues for further inquiries into multidimensional data is obvious.

Concerning the plant samples, the PCA models showed some separation of groups treated with different toxicants, but not as clearly as was the case with the PA cell suspension. Only the glyphosate-treated plants could be distinguished from the DCMU-treated ones. Also, our PCA models could not clearly differentiate between the concentrations of the same toxicant, although a certain dose-dependent trend was apparent in the distribution of the groups. The best separation was observed on the data from 72 h. This lack of discrimination between concentrations is consistent with the PCA models for individual toxicants as well as with the level of significant differences calculated by ANOVA.

Quantification of physiological processes in a non-destructive way by imaging techniques is a substantial advantage and a multi-sensor imaging equipment for an early-warning system to be used in agriculture and horticulture was proposed [6]. The wide array of stress detection methods compatible with the imaging approach including MCF and ChlF offers numerous possibilities for defining specific stress signatures.

PCA models make it easy to visualize and compare multi-dimensional data. PCA can also serve for selection of the most informative parameters and reduce the number of data dimensions prior to more detailed analyses.

In order to obtain precise and highly informative results, it is important to take into account the response time, the concentration and the set of informative parameters. Combination of parameters from different methods could be beneficial, as demonstrated also by our results where in some cases the Fv/Fm reacted earlier than MCF and even to lower concentrations.

## Conclusions

The PA cell suspension in combination with MCF and ChlF methods proved useful as a high-throughput pre-screening system of plant toxic stress. The advantages of using PA cell suspensions over whole plants for this purpose include high-throughput data acquisition, rapid stress detection, high sensitivity, homogenous stress response, and reduction in cultivation space, material and toxic compounds.

Having employed a combination of fluorescence parameters and PCA, we were able to detect differences at qualitative (different toxicants) as well as quantitative (different concentrations) levels. This suggests a potential of MCF and ChlF for a diagnostic use.

There is a prospect for application of our results in fields such as phenotyping, horticulture, ecotoxicology and basic stress-related research, especially at the cellular level with the use of PA cell suspensions. In the future, such signature approach based on diverse parameters could be used (a) in early stress detection; (b) phenotyping of tolerant or sensitive plant varieties; and (c) bring insight into the mode of action of unknown compounds or mixtures.

## Methods

### Plant material

The PA cell suspension of *Arabidopsis thaliana* was obtained from [74]. It was maintained under axenic conditions in Erlenmeyer flasks under 21 °C as described in [75]. The culture was sub-cultured every 3 weeks by transferring into fresh and autoclaved Gamborg medium (G 0210, Duchefa) with added 2,4-d (1 mg L^−1^), pH = 5.7. Actively growing suspension (7–8 days after subculturing) was used for experiments. The density of the suspension prior the toxicant application was adjusted to approx. 900 nephelometric units (measured with the OD Scanner, BugLab, US).

*Arabidopsis thaliana* Col-0 plants were used as the whole-plant material. Plants were grown one plant per pot in a cultivation chamber under 24 °C and 200 µmol m^−2^ s^−1^ photosynthetically active radiation with a 16/8 h light/dark photoperiod and 65% relative humidity. For experiments with chromium, the plants were grown hydroponically to ensure environmentally relevant root exposure and to avoid sorption or other interaction of the toxicant with soil particles. Four plants were planted per 0.5 L vessel filled with autoclaved half-strength Gamborg medium, pH = 5.7. Plants in a 2- to 4-true-leaf stage were used in the experiments according to [2].

### Growth monitoring

Routinely, cell suspension growth can be presented as change in packed cell volume, DW or turbidity [75]; however, these techniques require relatively large sample volumes. Therefore, we present a measure of viability inhibition (Table 1c). The viability was measured microscopically after staining with 0.4% water solution of Trypan blue (Carl Roth, DE) after [75]. The per cent viability inhibition is calculated as VI = (1−(x/c))*100, where VI is the viability inhibition, x is an average viability of the treated group, and c stands for average viability of the control group. The data for viability inhibition is available only for some of the tested concentrations, because these measurements were part of the pilot concentration-range-finding experiments which were conducted in higher volumes. Not all final concentrations were part of the pilot experiments, hence the missing values in Table 1c. The pilot experiments were repeated twice with comparable results. Presented are average data measured at 72 h.

The growth of the plants was measured in several ways. At each measured time point a projected leaf area was derived from an image taken by FluorCam to obtain an estimate of the rosette size in time. Also, the whole rosette DW was measured at 21 d as recommended by OECD [2]. Percent growth inhibition was calculated for each sample relative to average control in a way similar to the viability inhibition of PA cell suspension as GI = (1−(x/c)) * 100, where GI is the growth inhibition, x is DW of a sample at 21 d, c is average DW of control samples at 21 d.

### Experimental design

In order to verify the potential of the selected methods to capture different stress signatures, three toxicants with different and known modes of effect were chosen: (a) 3-(3,4-dichlorophenyl)-1,1-dimethylurea (DCMU), organic compound, a photosynthesis-inhibiting herbicide; (b) glyphosate, organic compound, a herbicide inhibiting aromatic amino acid biosynthesis; (c) potassium dichromate (chromium), inorganic compound (ion) with various negative effects including damage to pigments, enzymes and membranes via reactive oxygen species evolution [76]. The differences in their mode of action should be also exhibited as differences in ChlF and MCF parameters.

The experiments with PA cell suspension were conducted in translucent 96-well microplates. Firstly, a toxic solution was applied—100 µL per well—followed by addition of 100 µL of the PA cell suspension. The number of wells used per treatment was six to eight. The remaining wells were filled with water to decrease evaporation from the testing wells. The microplate was covered with plastic wrap and kept in a photoincubator on a shaker under cultivation conditions described above. The experiments were repeated three times with comparable results.

In the experiments with plants, DCMU and glyphosate were applied via a foliar spray. Chromium was applied to the roots by adding a concentrated solution to the cultivation medium, resulting in the desired working concentration. The number of plants per treatment was six in the case of foliar application (DCMU and glyphosate) and eight to 12 plants per treatment in the case of root application (chromium). The experiments were repeated two to four times with comparable results.

### Fluorescence imaging

The MCF and ChlF stress responses of the PA cell suspension were measured with an Open FluorCam FC 800-O/2020 and analyzed using the FluorCam7 software (Photon Systems Instruments, CZ). The MCF was acquired by a built-in MCF protocol using UV excitation light (385 nm) and four emission filters: blue (FF01-440/40), green (FF02-520/28), red (FF01-690/8), far-red (FF01-747/33). The Fv/Fm was derived from a built-in quenching analysis under orange-red measuring light (617 nm) and blue saturation pulse (450 nm, 1070 µmol m^−2^ s^−1^). In order to avoid complications with rapid sedimentation of the suspension cultures, the FluorCam was used in an inverted set-up as described in [75]. The measurements were made after 1.5, 6, 24 and 48 h. The samples treated with chromium were also measured at 72 h. Prior to measurements, the PA cell suspension in the microplates was pre-darkened for 10 min. Afterwards, measurement of ChlF followed by MCF was conducted.

The MCF and ChlF responses of plants were measured with an Open FluorCam FC 800-O as described in [77, 78]. The measurements were made after 1.5, 24, 72 and 120 h. The plants treated with glyphosate and chromium were also measured at 168 h. Prior to the measurements, the plants were pre-darkened for 30 min. Afterwards, the measurement of ChlF followed by MCF was conducted.

### Toxicants

The DCMU (Sigma-Aldrich, USA) was first dissolved in 80–90% ethanol to obtain a 50 mM stock solution. Afterwards it was diluted to desired concentrations with demineralized water. In the experiments with plants, the surfactant Triton X-100 (Sigma-Aldrich, USA) was added in a final concentration of 0.01%. The same concentration of ethanol and Triton X-100 (for plants) was used for mock-controls.

The glyphosate (Glyfosate 360 TF, Realchemie, NL) stock was diluted with demineralized water to reach the desired concentrations. The working solutions of chromium were prepared by dissolving potassium dichromate (Penta, CZ) in demineralized water. All stocks and working solutions were prepared fresh prior to the experiments. The final concentrations normalized to 1 g of DW are in Table 1, columns b, e. The accuracy of concentrations of the tested toxic solutions was approved by a comparison to standard solutions measured by HPLC–MS (DCMU and glyphosate) and ICP-MS (chromium); data not shown.

### Data analyses

Data preparation was done using Microsoft Excel for Mac 15.32 (Microsoft Corporation, Redmond, Washington, USA), statistical analyses and graphs were done using the R 3.5.1 software (R Core Team, Vienna, Austria).

The data sets obtained by measuring the MCF and Fv/Fm are of different scales (raw fluorescence signal, fluorescence ratios). For better visualization and comparison in spider graphs and for all PCA analyses, the data was normalized to average control values as n_norm_ = ((n/c)−1)*100, where n_norm_ is the normalized sample value, n is the sample value obtained by measurement, c is the average control value. As a result, each data point indicates a percentage difference from the average control value for a specific time point.

For PCA analyses the normalized data were further standardized to have mean zero and variance one. Only the parameters that did not mutually correlate (R^2^ < 0.8) and gave the best group separation in the model were selected for PCA. The ellipses in the PCA biplots show 95% confidence intervals.

The statistical significance of differences was tested using one-way ANOVA followed by a Tukey post hoc test. When the assumptions of normal distribution were not met, the Kruskal–Wallis test was used. In the case of violated assumption of homogeneity of variances, Welch’s ANOVA was used. The presented values are means with standard error and the differences are significant at p < 0.05.

## Supplementary information

**Additional file 1.** Additional line graphs, spider graphs, PCA biplots and table of PCA loadings for all tested concentrations and time points, visual effects on representative samples, flowchart of experimental design and scheme of data organization and inputs used for analyses.

## Data Availability

Datasets supporting the conclusions of this article are included within the article, its additional file and are available from the corresponding author on reasonable request.

## Abbreviations

ChlF: chlorophyll fluorescence
DCMU: 3-(3,4-dichlorophenyl)-1,1-dimethylurea
DW: dry weight
Fv/Fm: maximum quantum efficiency of photosystem II
F440: blue fluorescence
F520: green fluorescence
F680: red fluorescence
F740: far-red fluorescence
MCF: multicolor fluorescence
PA: photoautotrophic
PCA: principal component analysis
PC1: the first principal component
