# Supplementary material for: Noninvasive determination of toxic stress biomarkers by high-throughput screening of photoautotrophic cell suspension cultures with multicolor fluorescence imaging
Source: Plant Methods. 2019 Aug 24;15:100. doi: 10.1186/s13007-019-0484-y (PMC6708129; doi:10.1186/s13007-019-0484-y)
Supplement: Supplementary file 1 — Additional file 1. Additional line graphs, spider graphs, PCA biplots and table of PCA loadings for all tested concentrations and time points, visual effects on representative samples, flowchart of experimental design and scheme of data organization and inputs used for analyses. [file 13007_2019_484_MOESM1_ESM.pdf]

ADDITIONAL FILE 1

Contents:

Fig. S1 DCMU fluorescence signatures, dose- and time-dependency

Fig. S2 Glyphosate fluorescence signatures, dose- and time-dependency

Fig. S3 Chromium fluorescence signatures, dose- and time-dependency

Fig. S4 Time- and dose-dependent changes in PA cell suspension of *Arabidopsis thaliana* treated with DCMU

Fig. S5 Time- and dose-dependent changes in *Arabidopsis thaliana* plants treated with DCMU

Fig. S6 Time- and dose-dependent changes in PA cell suspension of *Arabidopsis thaliana* treated with glyphosate

Fig. S7 Time- and dose-dependent changes in *Arabidopsis thaliana* plants treated with glyphosate

Fig. S8 Time- and dose-dependent changes in PA cell suspension of *Arabidopsis thaliana* treated with chromium

Fig. S9 Time- and dose-dependent changes in *Arabidopsis thaliana* plants treated with chromium

Fig. S10 PCA models for DCMU, time-dependency

Fig. S11 PCA models for glyphosate, time-dependency

Fig. S12 PCA models for chromium, time-dependency

Fig. S13 PCA models for suspension, all tested toxicants, time-dependency

Fig. S14 PCA models for plants, all tested toxicants, time-dependency

Fig. S15 Visual effects of tested compounds on PA suspension

Fig. S16 Visual effects of DCMU on plants

Fig. S17 Visual effects of glyphosate on plants

Fig. S18 Visual effects of chromium on plants

Table S1 Summary table of PCA models

Fig. S19 Flowchart of experimental design

Fig. S20 Scheme of data organization and inputs used for analyses

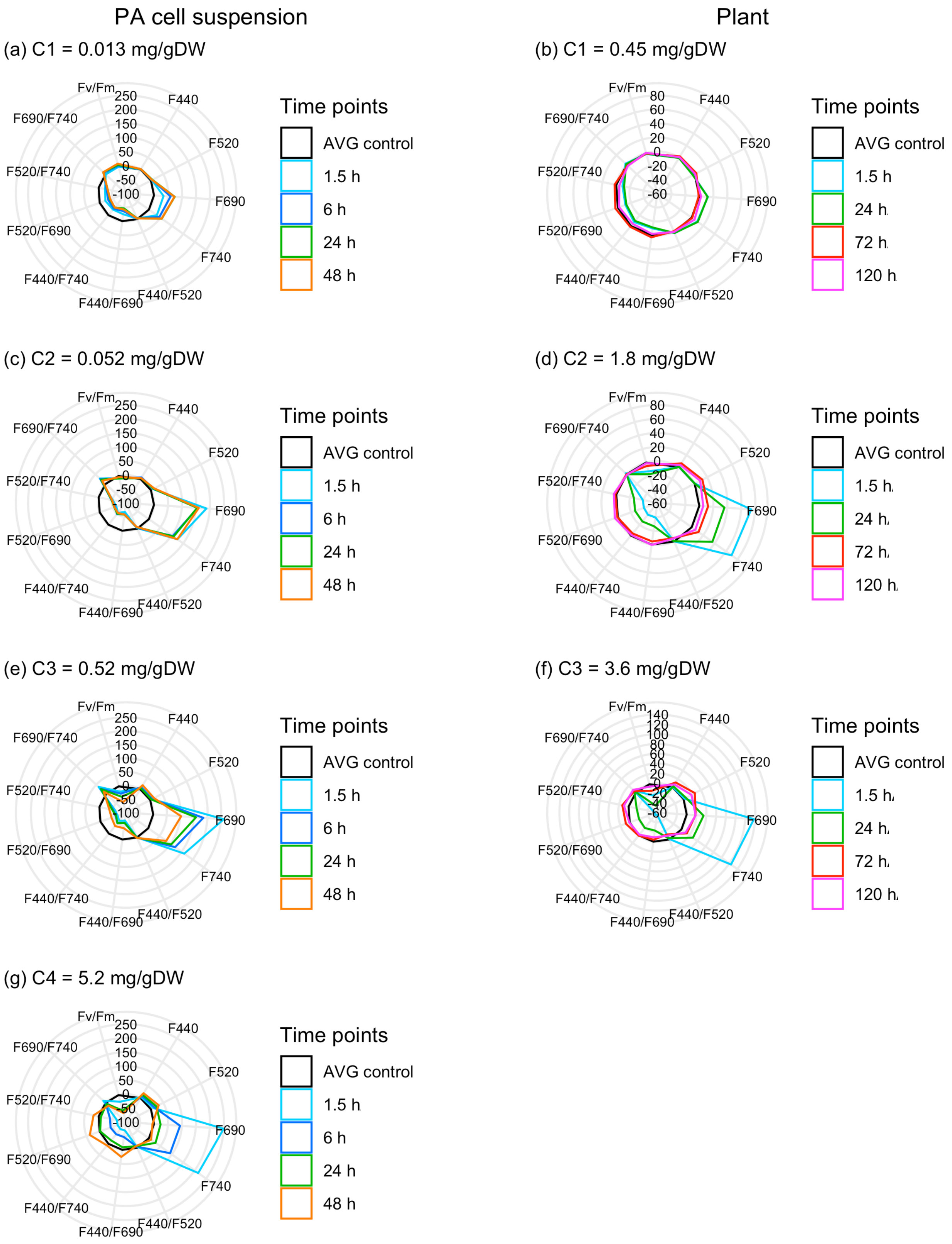

Fig. S1 DCMU fluorescence signatures, dose- and time-dependency. *Arabidopsis thaliana* PA cell suspension (left column) and plants (right column) treated with tested concentrations of DCMU. Data were normalized to average control in order to present percentage changes in parameter values. Presented are the mean values.

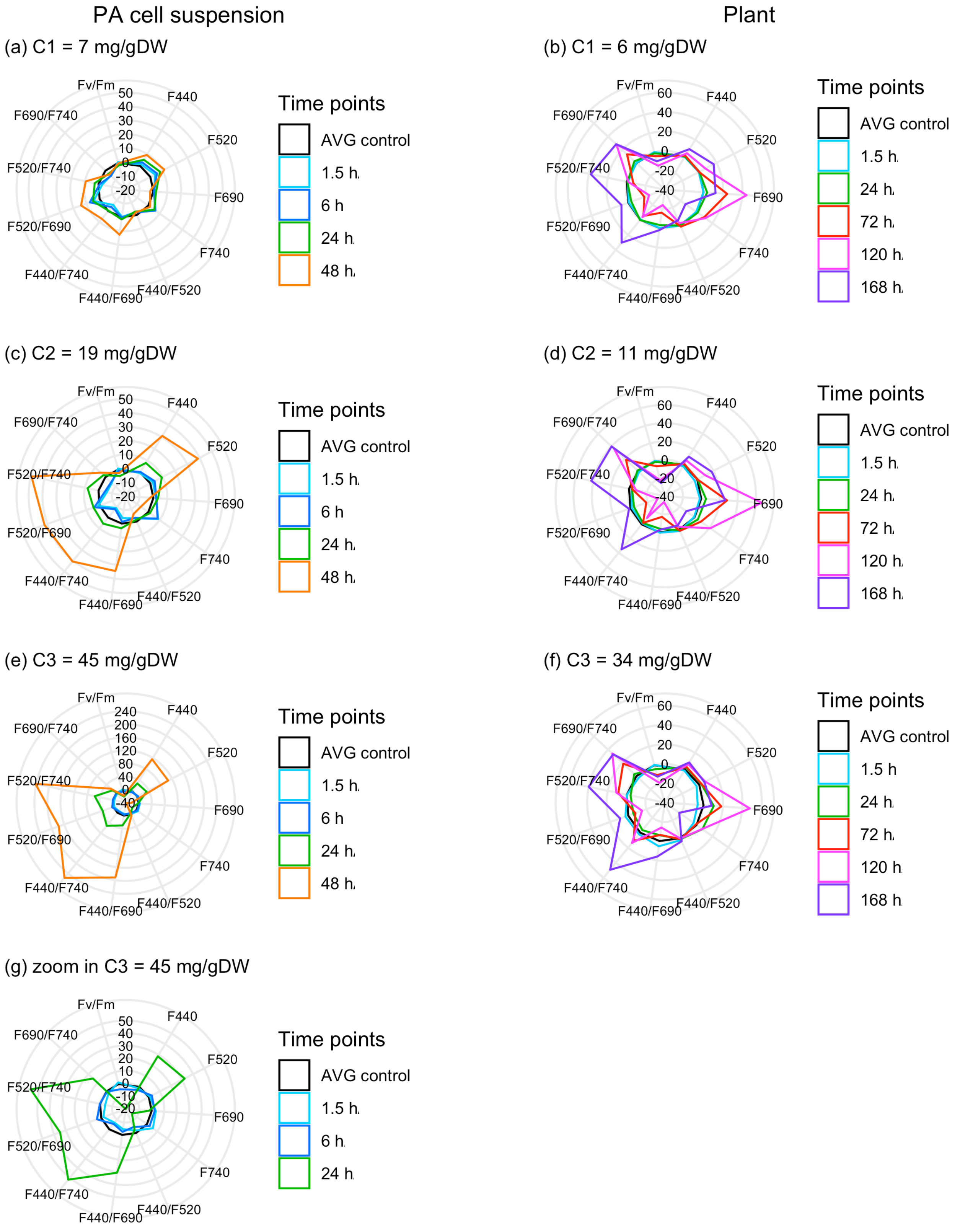

Fig. S2 Glyphosate fluorescence signatures, dose- and time-dependency. *Arabidopsis thaliana* PA cell suspension (left column) and plants (right column) treated with tested concentrations of glyphosate. Data were normalized to average control in order to present percentage changes in parameter values. Presented are the mean values.

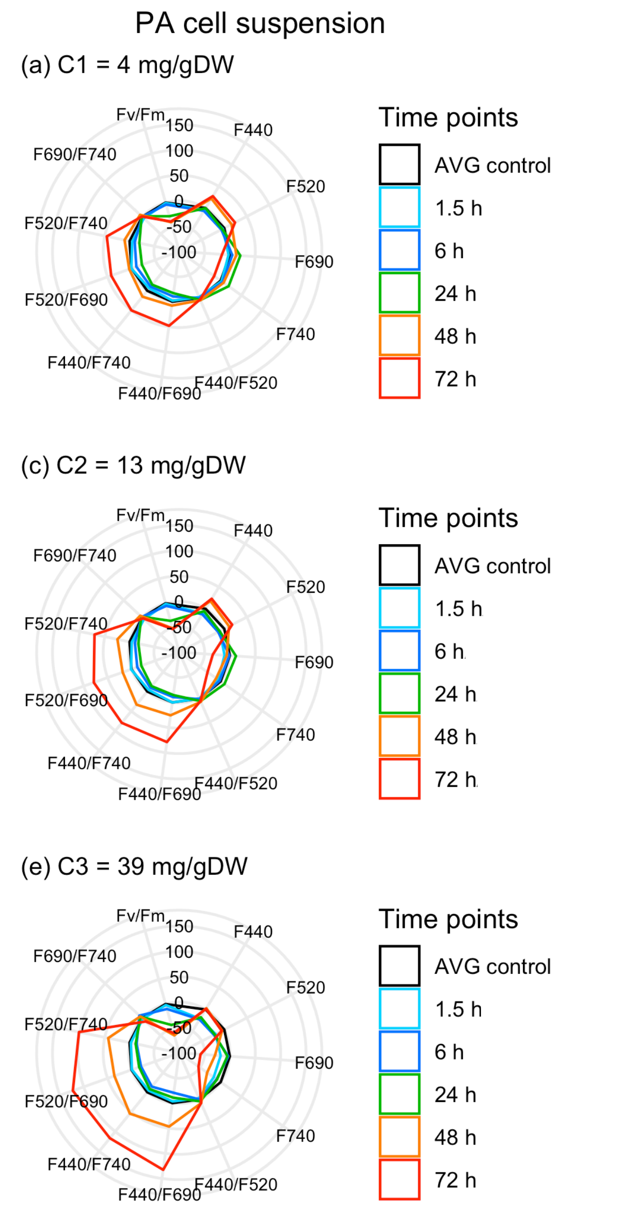

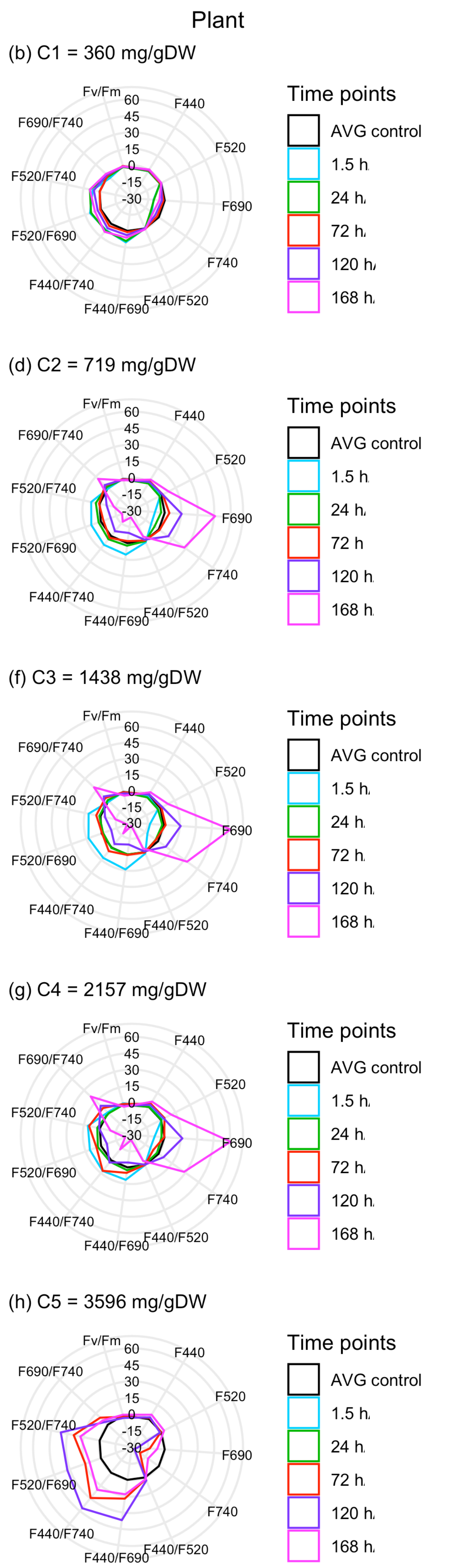

Fig. S3 Chromium fluorescence signatures, dose- and time-dependency. *Arabidopsis thaliana* PA cell suspension (left column) and plants (right column) treated with tested concentrations of chromium. Data were normalized to average control in order to present percentage changes in parameter values. Presented are the mean values.

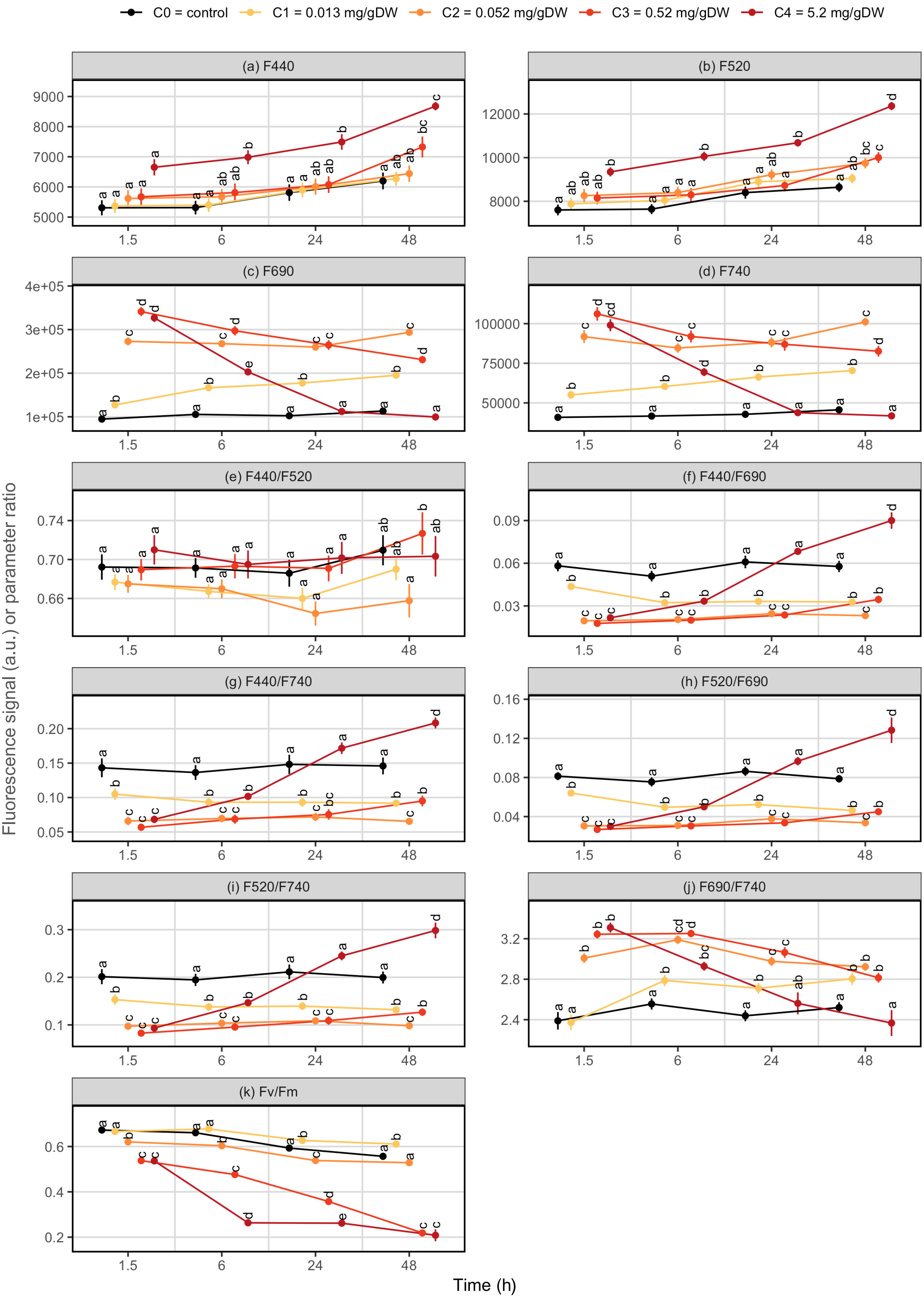

Fig. S4 Time- and dose-dependent changes in PA cell suspension of *Arabidopsis thaliana* treated with DCMU. Presented are measured parameters and ratios of multicolor fluorescence and Fv/Fm. Data are presented as means ± SE. Different letters indicate significant differences between groups at a specific timepoint ($p < 0.05$).

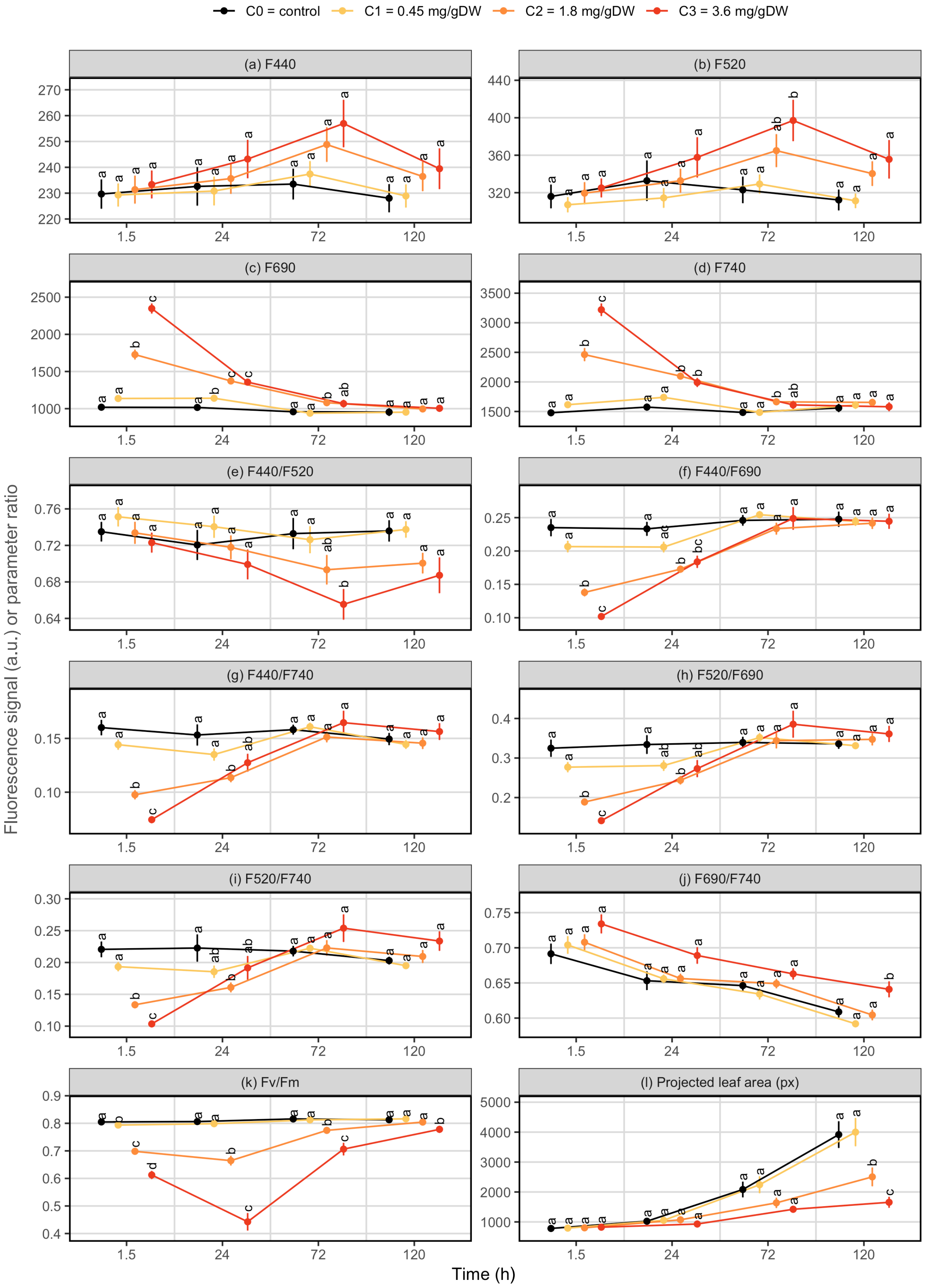

Fig. S5 Time- and dose-dependent changes in *Arabidopsis thaliana* plants treated with DCMU. Presented are measured parameters and ratios of multicolor fluorescence, Fv/Fm and projected rosette area. Data are presented as means ± SE. Different letters indicate significant differences between groups at a specific timepoint ($p < 0.05$).

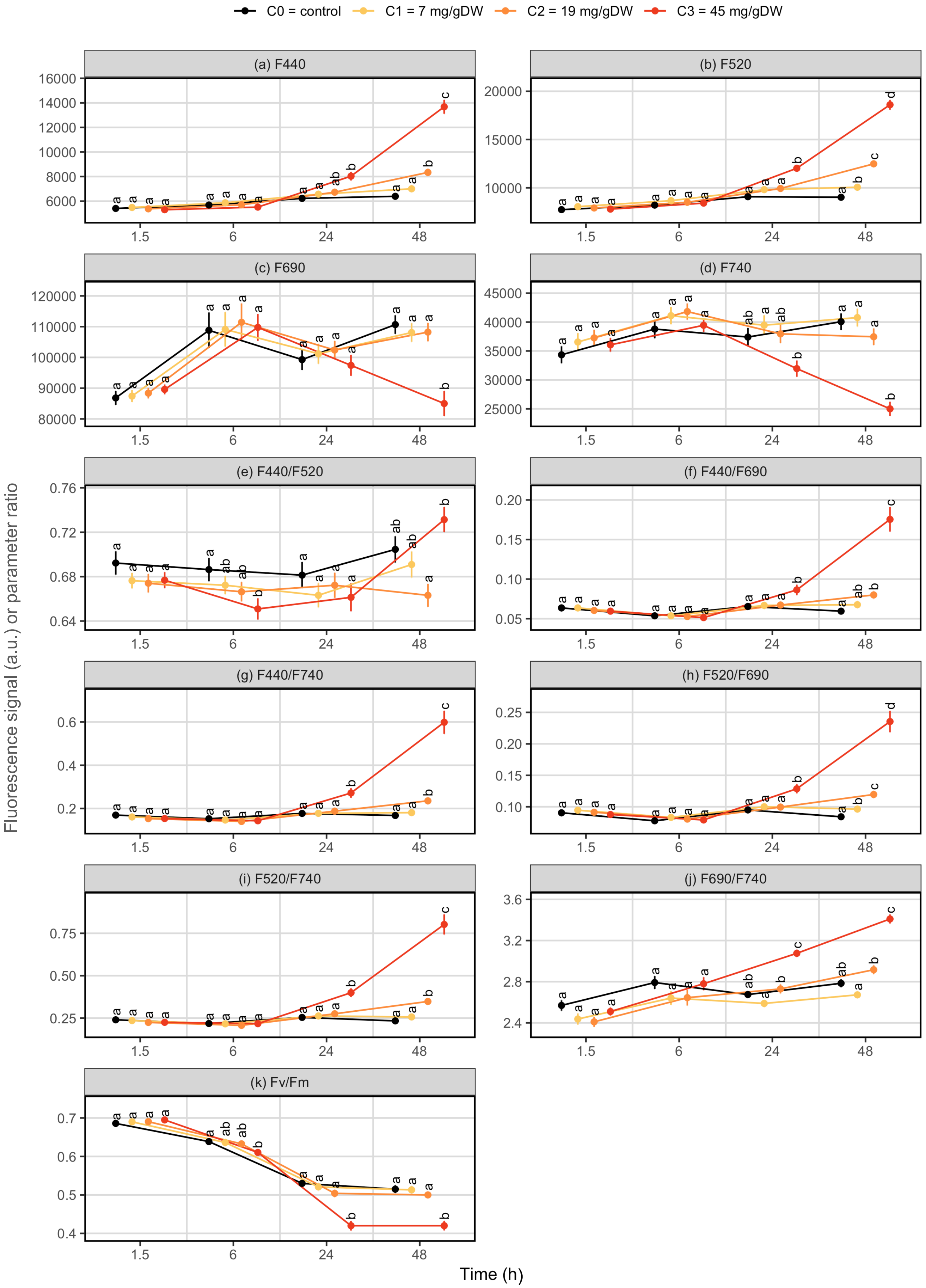

Fig. S6 Time- and dose-dependent changes in PA cell suspension of *Arabidopsis thaliana* treated with glyphosate. Presented are measured parameters and ratios of multicolor fluorescence and Fv/Fm. Data are presented as means ± SE. Different letters indicate significant differences between groups at a specific timepoint ($p < 0.05$).

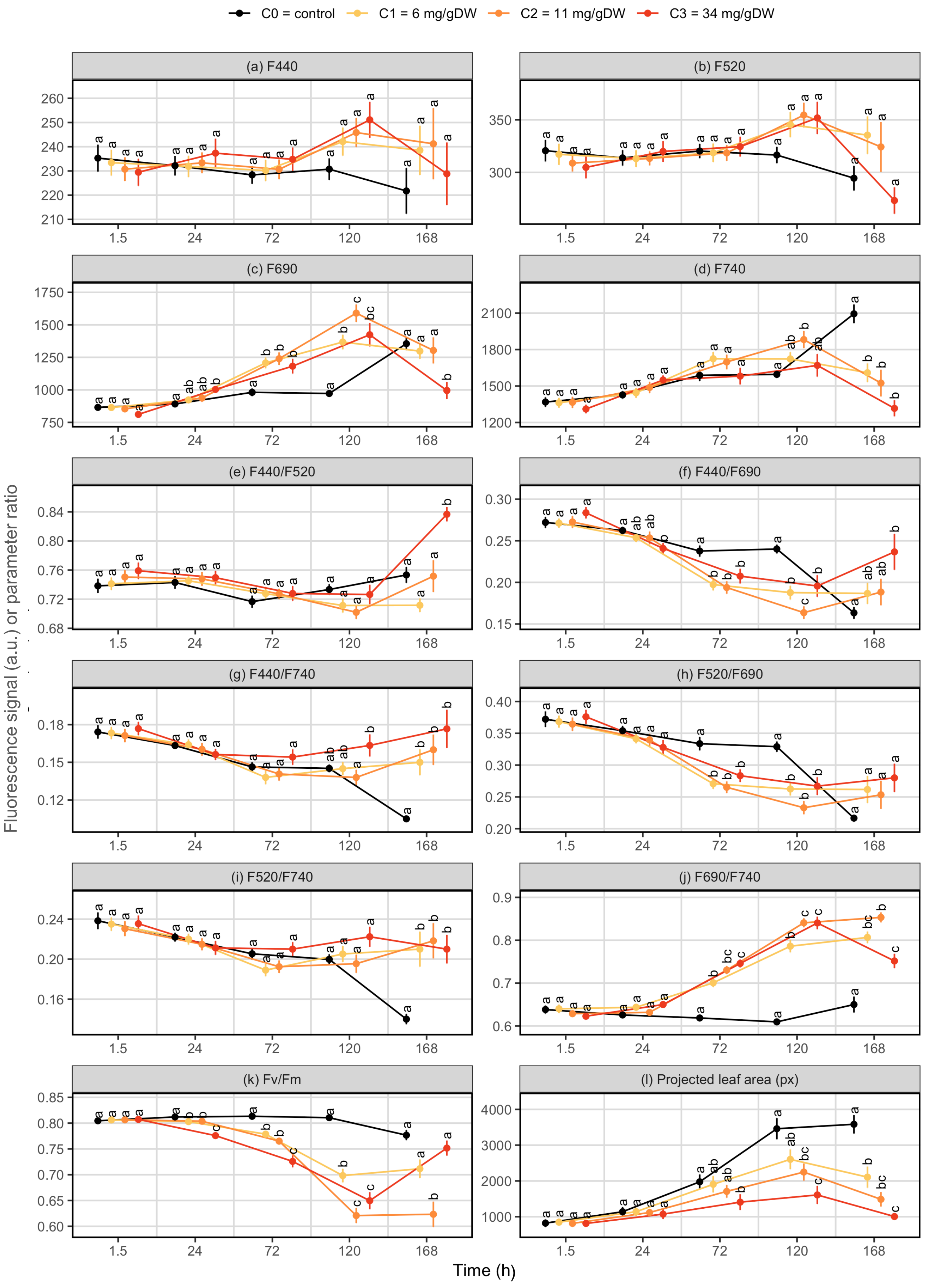

Fig. S7 Time- and dose-dependent changes in *Arabidopsis thaliana* plants treated with glyphosate. Presented are measured parameters and ratios of multicolor fluorescence, Fv/Fm and projected rosette area. Data are presented as means ± SE. Different letters indicate significant differences between groups at a specific timepoint ($p < 0.05$).

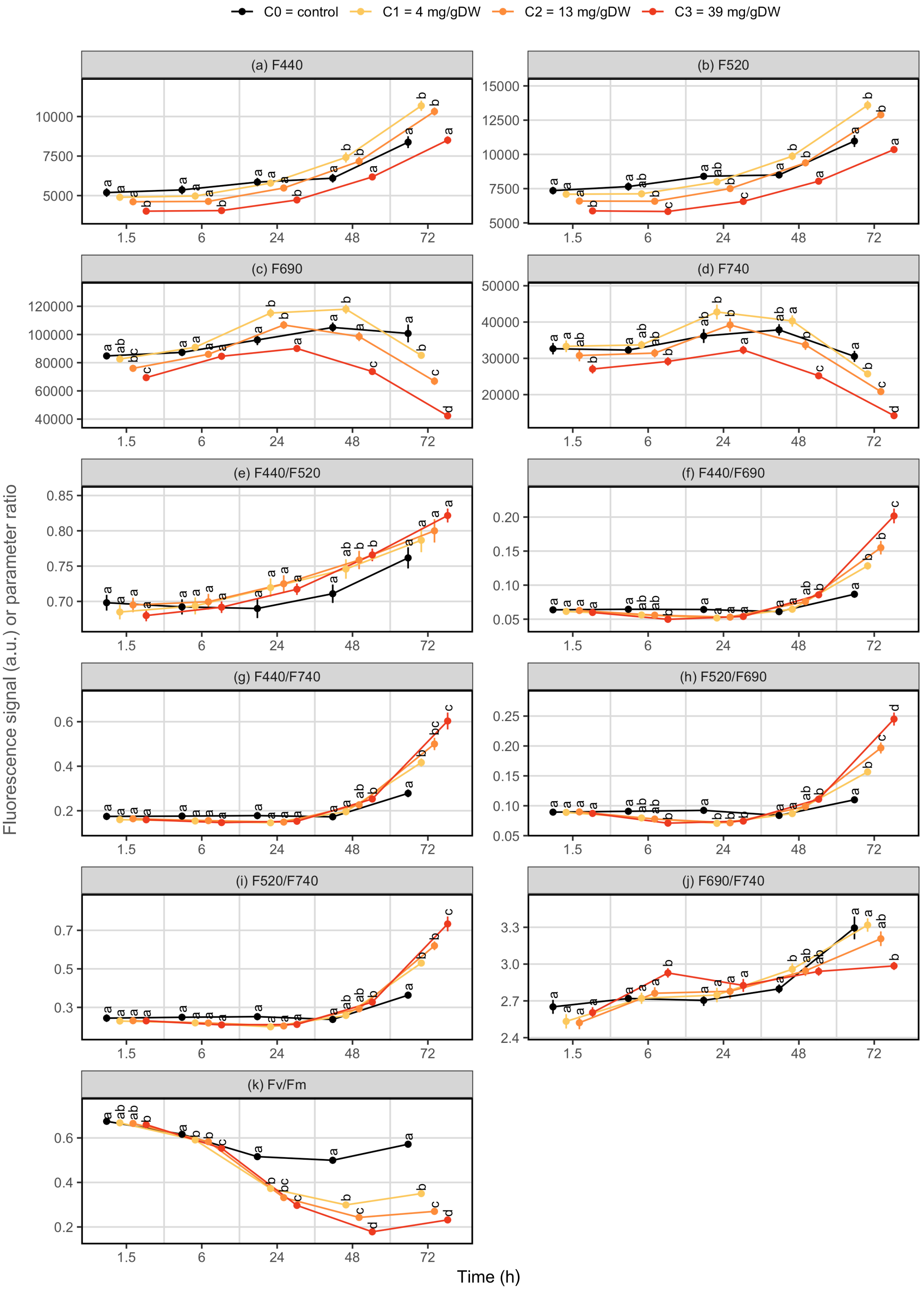

Fig. S8 Time- and dose-dependent changes in PA cell suspension of *Arabidopsis thaliana* treated with chromium. Presented are measured parameters and ratios of multicolor fluorescence and Fv/Fm. Data are presented as means ± SE. Different letters indicate significant differences between groups at a specific timepoint ($p < 0.05$).

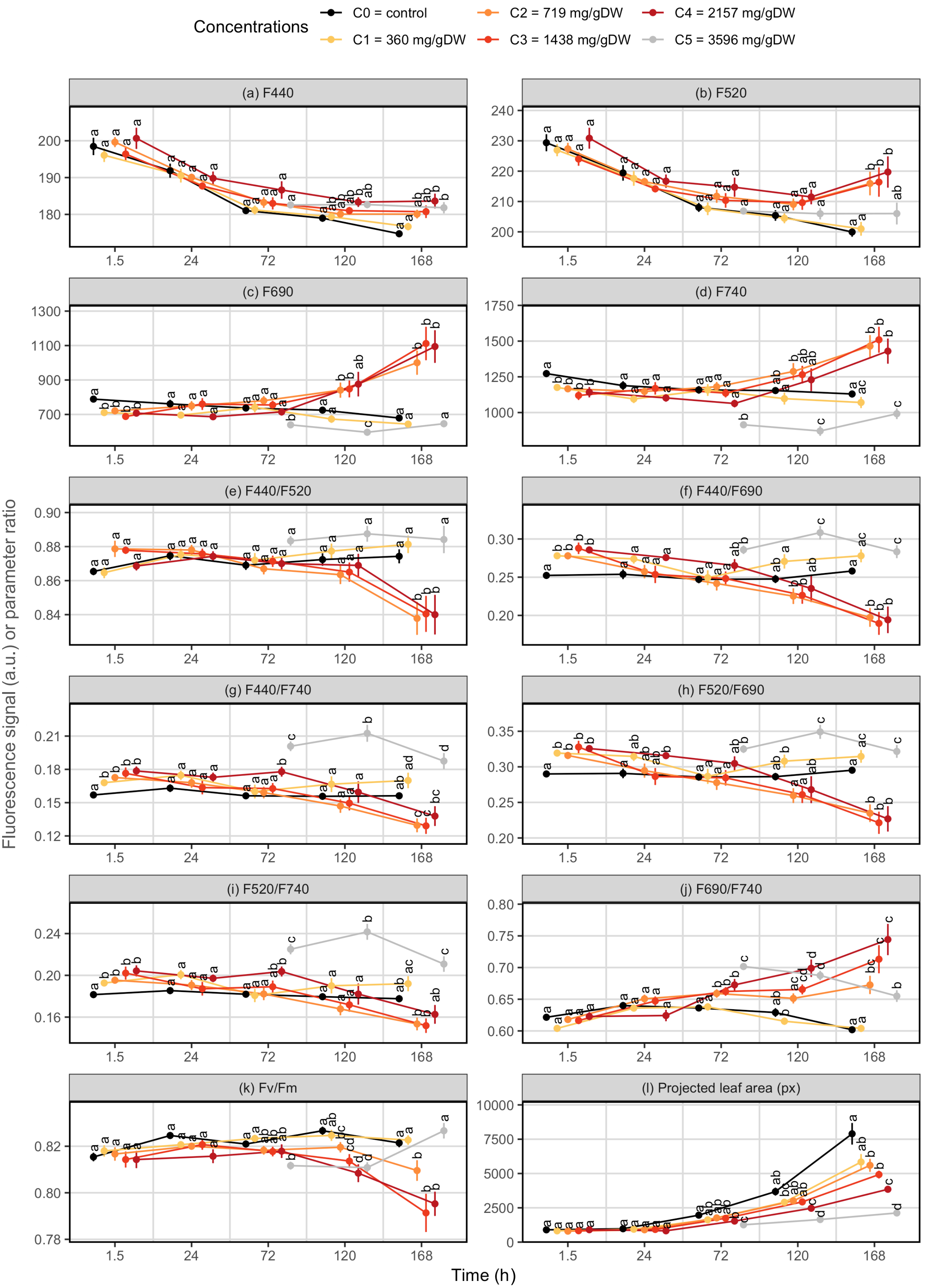

Fig. S9 Time- and dose-dependent changes in *Arabidopsis thaliana* plants treated with chromium. Presented are measured parameters and ratios of multicolor fluorescence, Fv/Fm and projected rosette area. Data are presented as means ± SE. Different letters indicate significant differences between groups at a specific timepoint ($p < 0.05$).

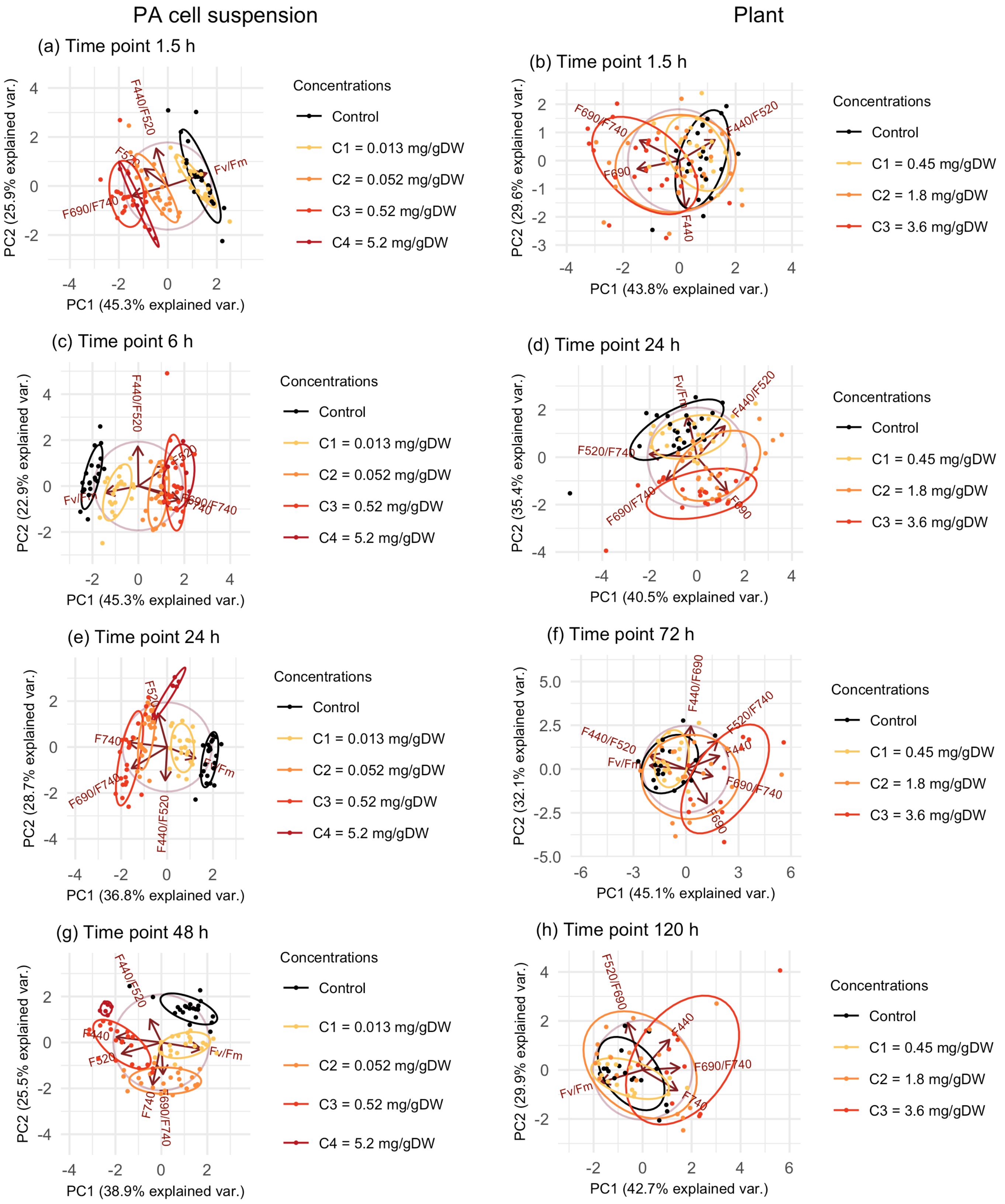

Fig. S10 PCA models for DCMU, time-dependency. The graphs show the discrimination between concentrations of DCMU treated *Arabidopsis thaliana* PA cell suspension (left column) and plants (right column) with PCA. Separation of the groups is shown for each tested time point.

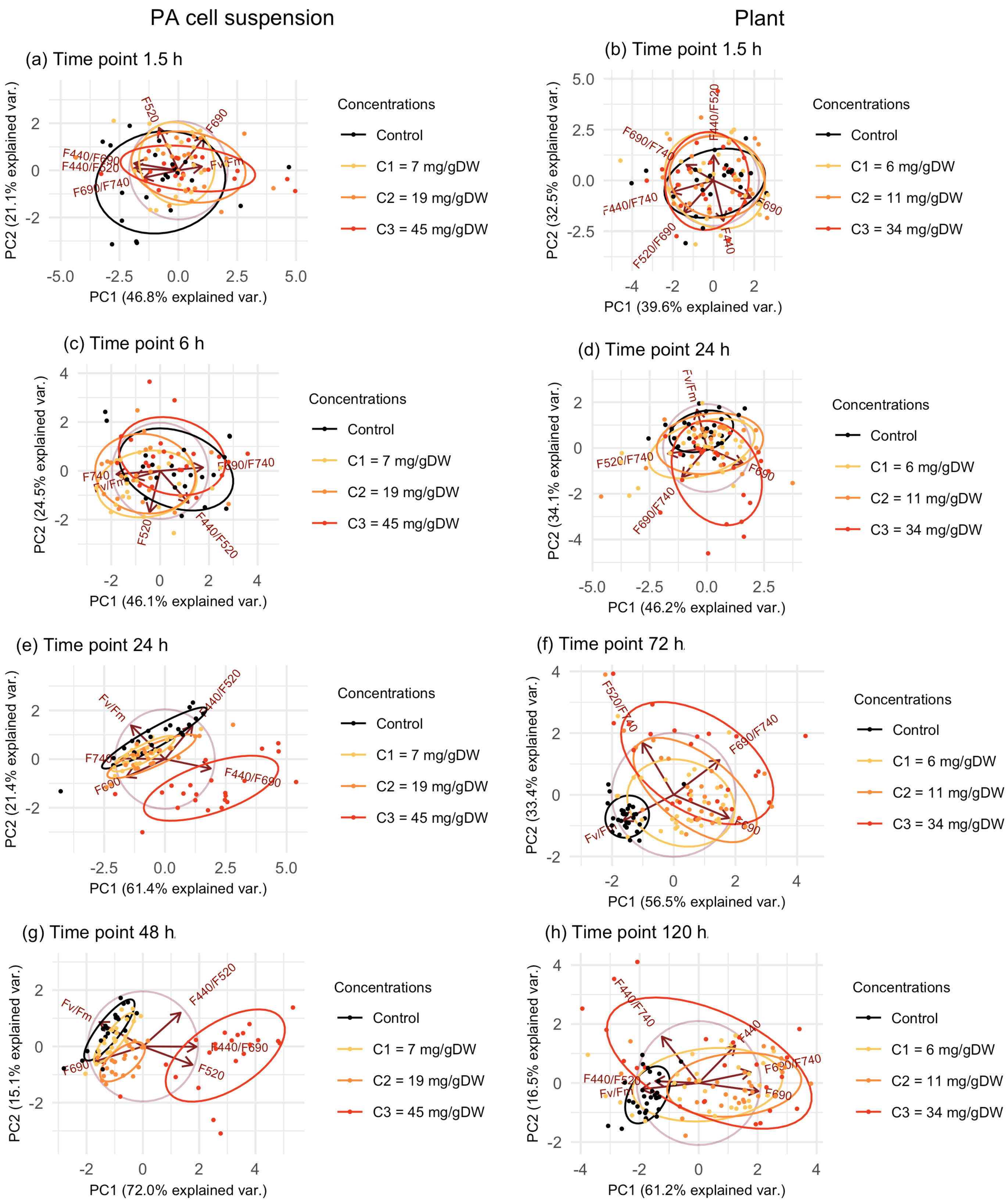

Fig. S11 PCA models for glyphosate, time-dependency. The graphs show the discrimination between concentrations of glyphosate treated *Arabidopsis thaliana* PA cell suspension (left column) and plants (right column) with PCA. Separation of the groups is shown for each tested time point.

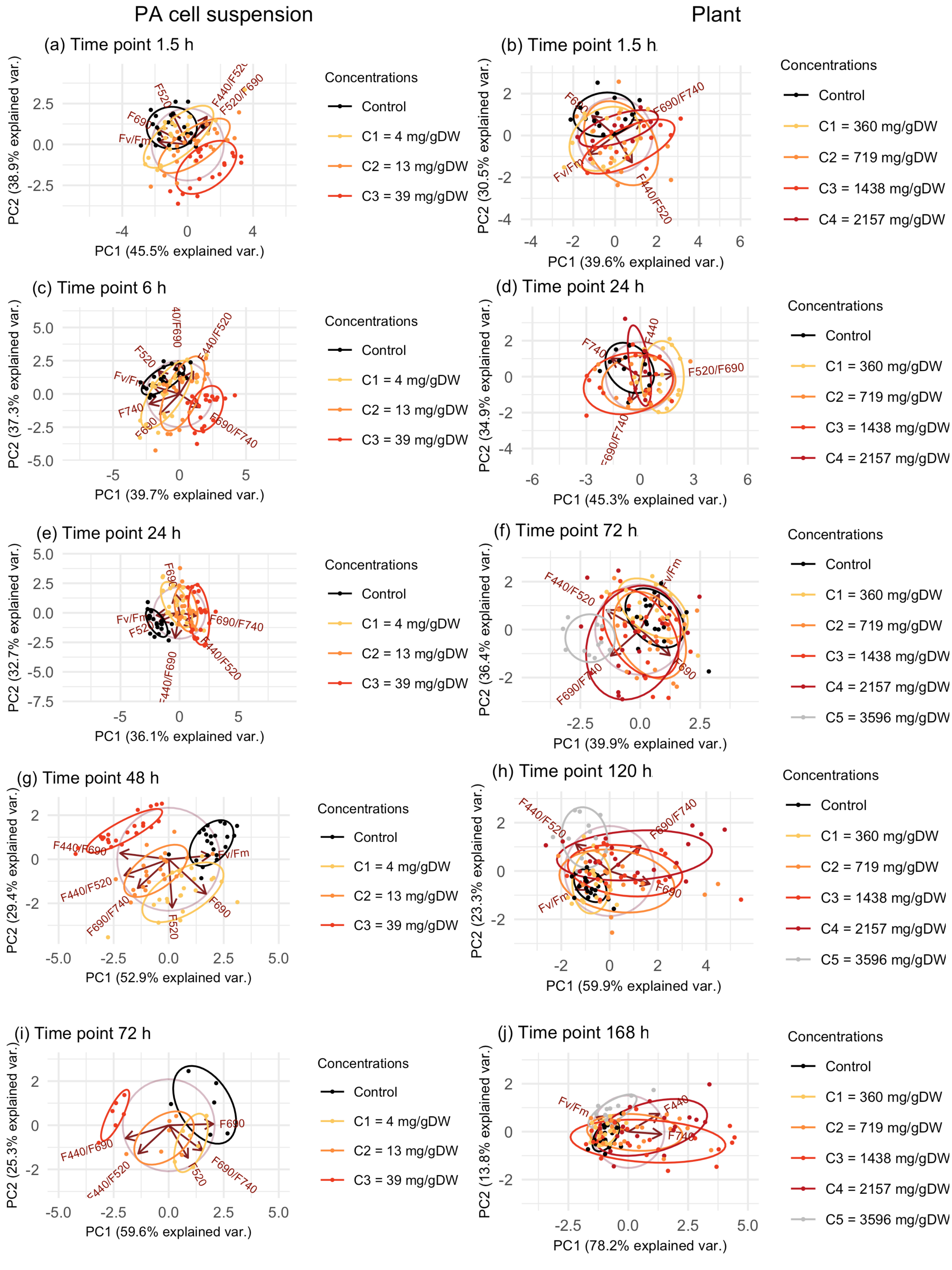

Fig. S12 PCA models for chromium, time-dependency. The graphs show the discrimination between concentrations of chromium treated *Arabidopsis thaliana* PA cell suspension (left column) and plants (right column) with PCA. Separation of the groups is shown for each tested time point.

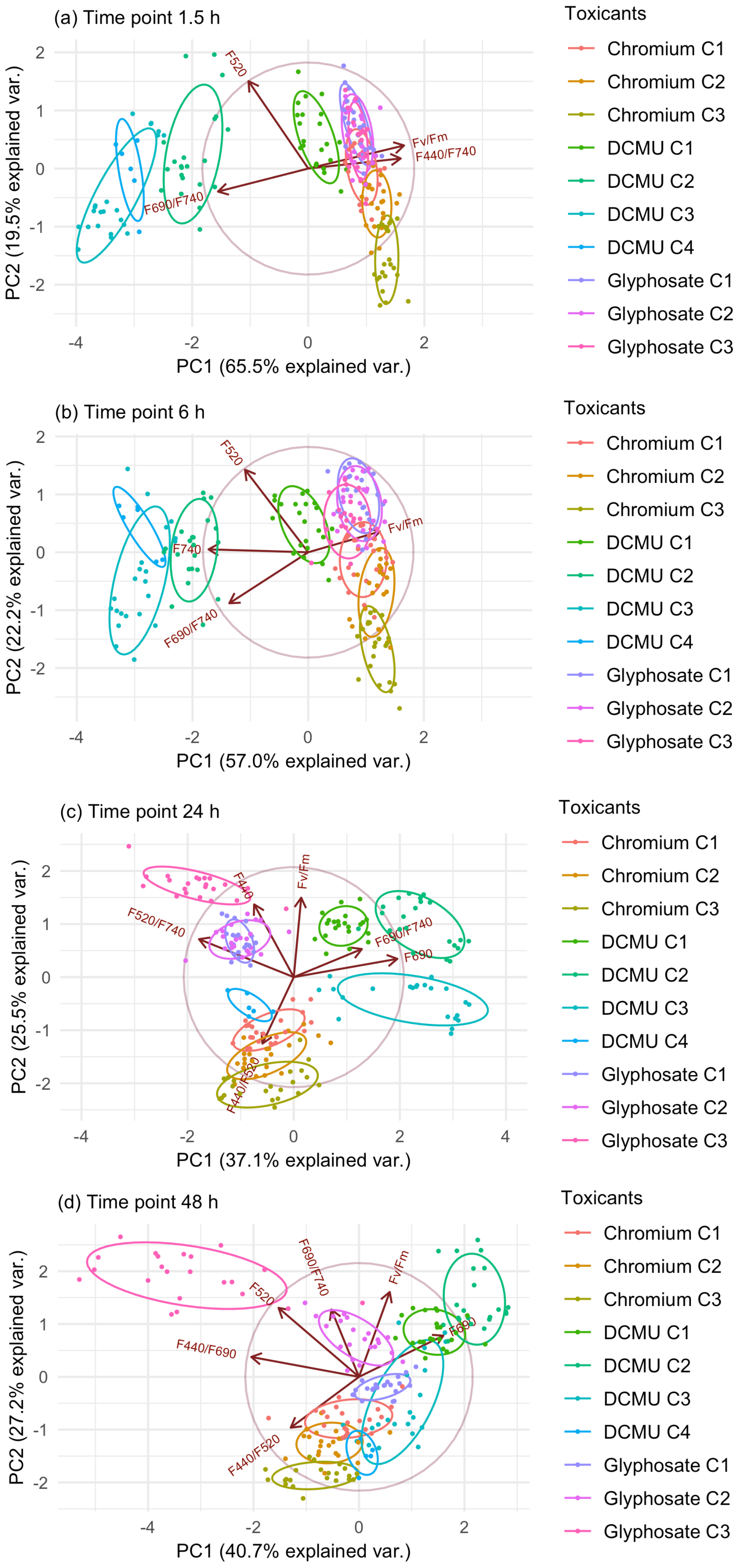

Fig. S13 PCA models for PA cell suspension, all tested toxicants, time-dependency. The graphs show the discrimination between concentrations of DCMU, glyphosate or chromium treated *Arabidopsis thaliana* PA cell suspension. Separation of the groups is shown for each tested time point.

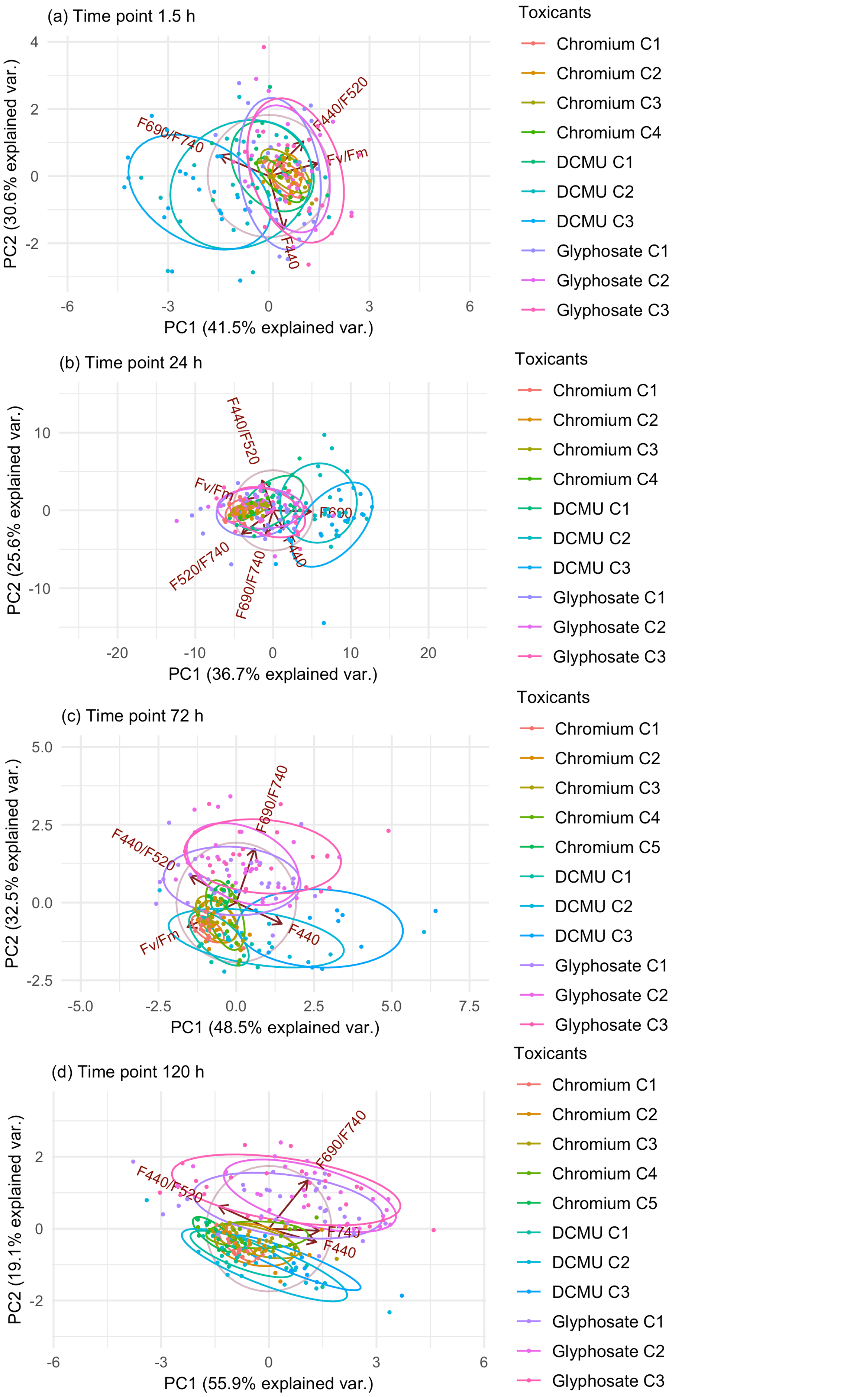

Fig. S14 PCA models for plants, all tested toxicants, time-dependency. The graphs show the discrimination between concentrations of DCMU, glyphosate or chromium treated *Arabidopsis thaliana* plants. Separation of the groups is shown for each tested time point.

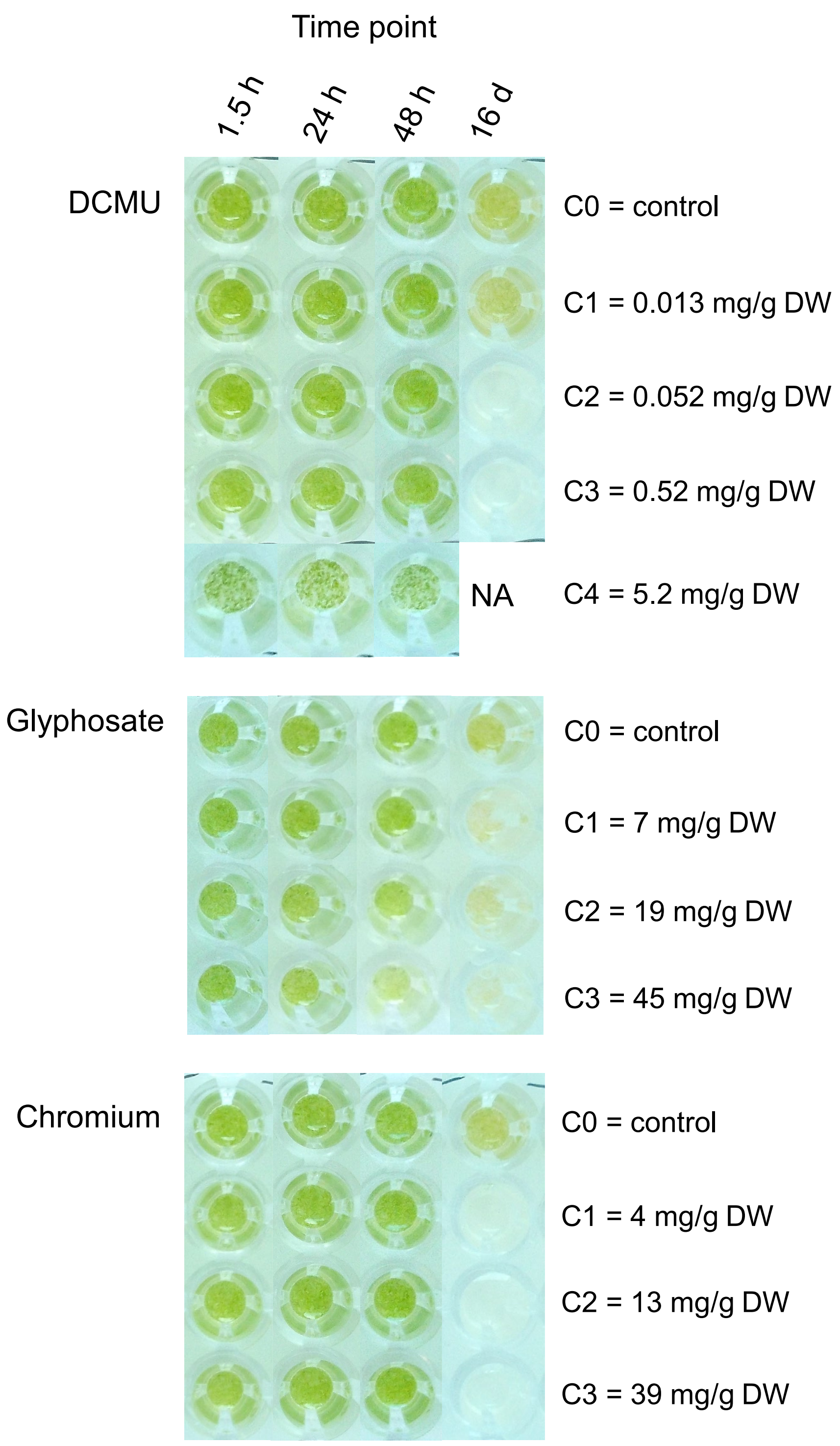

Fig. S15 Visual effects of tested compounds. Representative samples of *Arabidopsis thaliana* PA cell suspension. NA = not available.

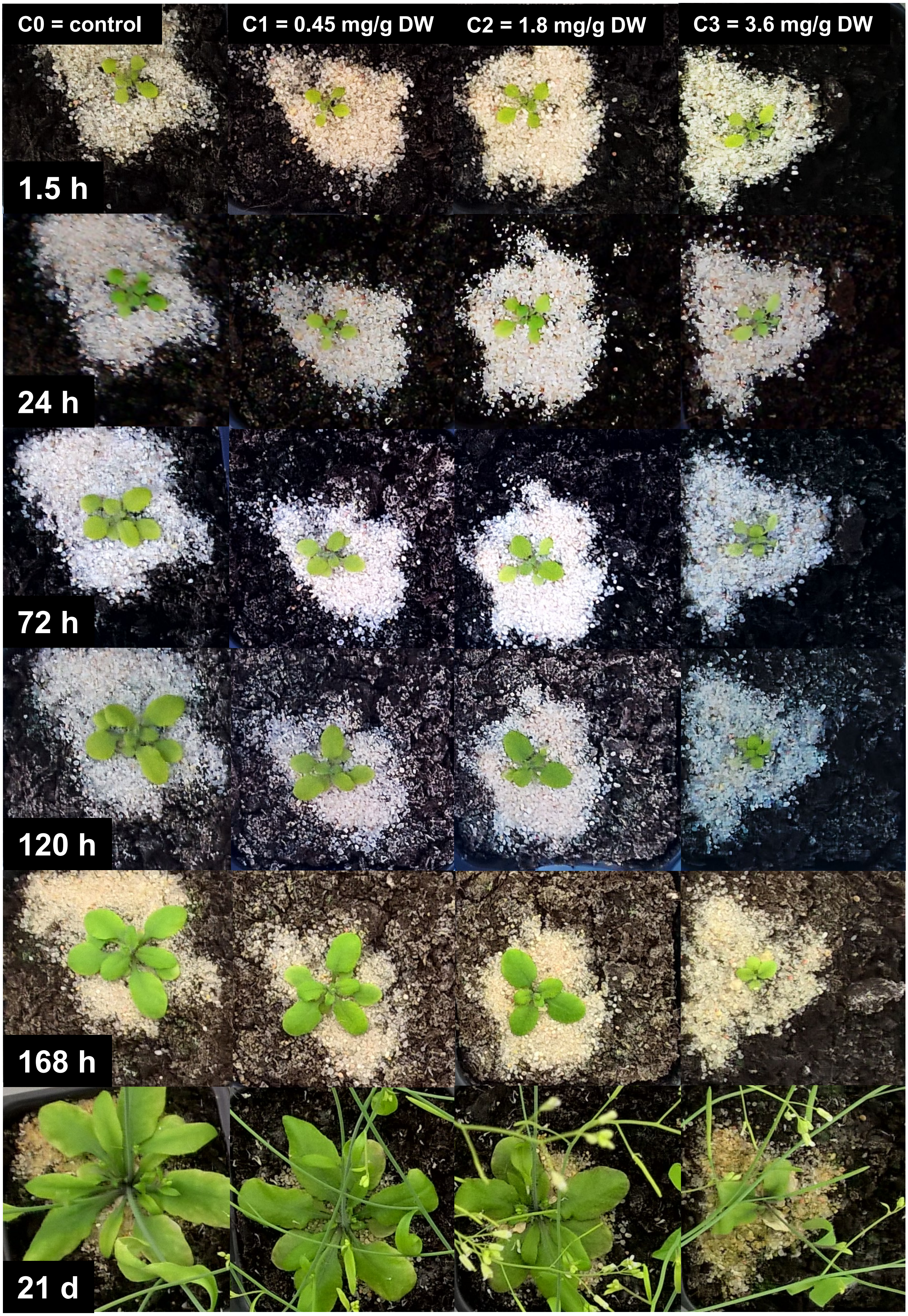

Fig. S16 Visual effects of DCMU. Representative samples of *Arabidopsis thaliana* plants. The sand was used to cover algal growth on soil interfering with the fluorescence signal of the plants.

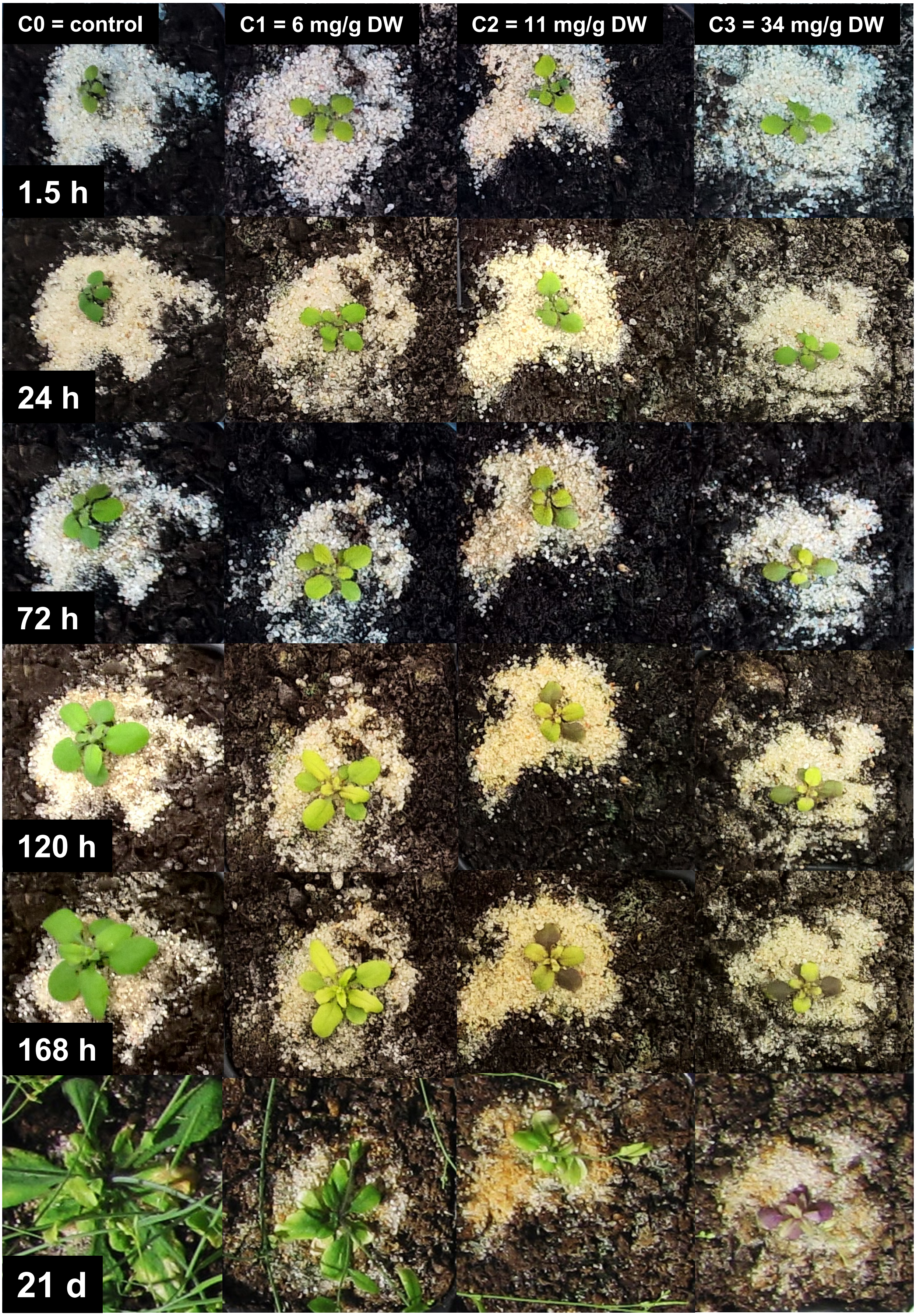

Fig. S17 Visual effects of glyphosate. Representative samples of *Arabidopsis thaliana* plants. The sand was used to cover algal growth on soil interfering with the fluorescence signal of the plants.

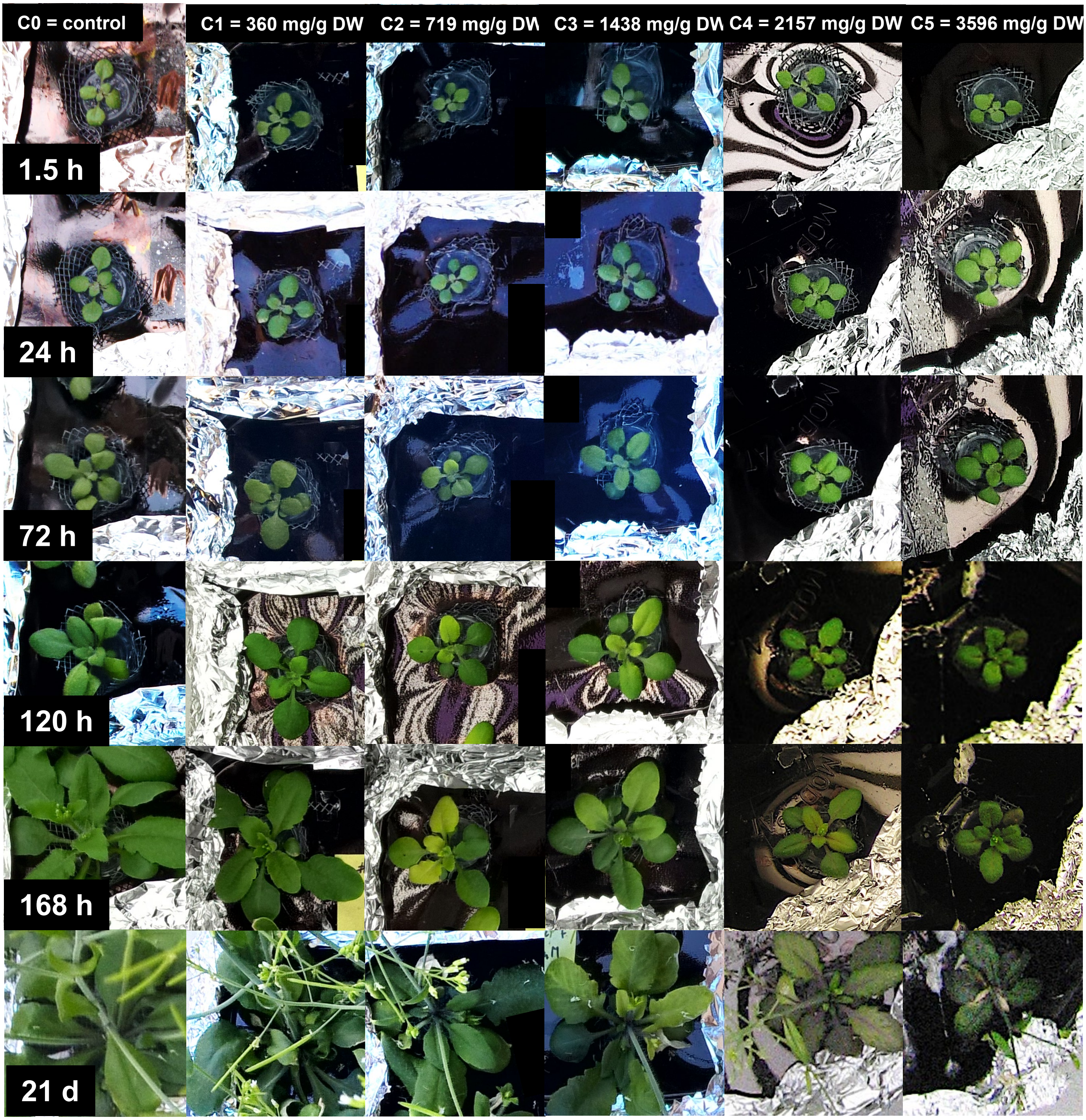

Fig. S18 Visual effects of chromium. Representative samples of *Arabidopsis thaliana* plants grown hydroponically.

Table S1 Summary table of PCA models.

time point (h)			1.5			6			24			48			72	
			PC1	PC2		PC1	PC2		PC1	PC2		PC1	PC2		PC1	PC2
PA cell suspension		eigenvalue	1.81	1.03		2.26	1.15		1.84	1.43		2.33	1.53			
		explained variance (cummul.)	0.45	0.71		0.45	0.68		0.37	0.65		0.40	0.64		NA	
		variables	variable importance		variables	variable importance		variables	variable importance		variables	variable importance		variables	variable importance	
	DCMU	F520	- 0.40	0.39	F520	0.44	0.42	F520	- 0.15	0.63	F440	- 0.61	0.09			
		F440/F520	- 0.20	0.85	F740	0.61	- 0.29	F740	- 0.64	0.09	F520	- 0.54	- 0.19			
		F690/F740	- 0.60	- 0.22	F440/F520	- 0.01	0.83	F440/F520	- 0.03	- 0.63	F740	- 0.12	- 0.71		NA	
		Fv/Fm	0.66	0.29	F690/F740	0.43	- 0.15	F690/F740	- 0.59	- 0.40	F440/F520	- 0.15	0.39			
					Fv/Fm	- 0.50	- 0.14	Fv/Fm	0.47	- 0.21	F690/F740	0.02	- 0.53			
											Fv/Fm	0.54	- 0.11			
			PC1	PC2		PC1	PC2		PC1	PC2		PC1	PC2		PC1	PC2
		eigenvalue	2.80	1.27		2.31	1.22		3.07	1.07		3.60	0.76			
		explained variance (cummul.)	0.47	0.68		0.46	0.71		0.61	0.83		0.72	0.87		NA	
		variables	variable importance		variables	variable importance		variables	variable importance		variables	variable importance		variables	variable importance	
	glyphosate	F520	- 0.24	0.77	F520	- 0.14	- 0.78	F690	- 0.44	- 0.35	F520	0.48	- 0.38			
		F690	0.32	0.61	F740	- 0.60	- 0.06	F740	- 0.53	0.01	F690	- 0.46	- 0.31			
		F440/F520	- 0.54	0.03	F440/F520	0.39	- 0.60	F440/F520	0.32	0.65	F440/F520	0.36	0.71		NA	
		F440/F690	- 0.54	0.13	F690/F740	0.59	0.06	F440/F690	0.52	- 0.18	F440/F690	0.51	- 0.01			
		F690/F740	- 0.42	- 0.13	Fv/Fm	- 0.35	- 0.13	Fv/Fm	- 0.39	0.65	Fv/Fm	- 0.42	0.51			
		Fv/Fm	0.29	0.07												
			PC1	PC2		PC1	PC2		PC1	PC2		PC1	PC2		PC1	PC2
		eigenvalue	2.27	1.95		2.78	2.61		2.17	1.96		3.17	1.76		2.98	1.26
		explained variance (cummul.)	0.45	0.84		0.40	0.77		0.36	0.69		0.53	0.82		0.60	0.85
		variables	variable importance		variables	variable importance		variables	variable importance		variables	variable importance		variables	variable importance	
	chromium	F520	- 0.29	0.63	F520	- 0.38	0.43	F520	- 0.53	- 0.24	F520	0.04	- 0.71	F520	0.24	- 0.63
		F690	- 0.56	0.31	F690	- 0.36	- 0.38	F690	- 0.11	0.52	F690	0.41	- 0.51	F690	0.56	0.02
		F440/F520	0.35	0.55	F740	- 0.54	- 0.19	F440/F520	0.37	- 0.42	F440/F520	- 0.48	- 0.24	F440/F520	- 0.38	- 0.55
		F520/F690	0.44	0.44	F440/F520	0.25	0.42	F440/F690	- 0.12	- 0.69	F440/F690	- 0.53	0.09	F440/F690	- 0.54	- 0.25
		Fv/Fm	- 0.53	0.06	F440/F690	- 0.02	0.61	F690/F740	0.44	- 0.08	F690/F740	- 0.34	- 0.42	F690/F740	0.43	- 0.49
					F690/F740	0.37	- 0.25	Fv/Fm	- 0.61	- 0.07	Fv/Fm	0.45	0.05			
					Fv/Fm	- 0.48	0.15									
			PC1	PC2		PC1	PC2		PC1	PC2		PC1	PC2		PC1	PC2
		eigenvalue	2.62	0.78		2.28	0.89		2.22	1.53		2.44	1.63			
		explained variance (cummul.)	0.65	0.85		0.57	0.79		0.37	0.63		0.41	0.68		NA	
		variables	variable importance		variables	variable importance		variables	variable importance		variables	variable importance		variables	variable importance	
	pooled data for all toxicants	F520	- 0.35	0.93	F520	- 0.40	0.83	F440	- 0.24	0.53	F520	- 0.45	0.47			
		F440/F740	0.54	0.11	F740	- 0.63	0.03	F690	0.63	0.13	F690	0.47	0.28			
		F690/F740	- 0.53	- 0.25	F690/F740	- 0.50	- 0.51	F440/F520	- 0.19	- 0.49	F440/F520	- 0.38	- 0.35		NA	
		Fv/Fm	0.56	0.25	Fv/Fm	0.45	0.21	F520/F740	- 0.58	0.28	F440/F690	- 0.61	0.14			
								F690/F740	0.41	0.21	F690/F740	- 0.16	0.46			
								Fv/Fm	0.05	0.58	Fv/Fm	0.17	0.58			
time point (h)			1.5			24			72			120			168	
			PC1	PC2		PC1	PC2		PC1	PC2		PC1	PC2		PC1	PC2
plant		eigenvalue	1.75	1.18		2.02	1.77		3.16	2.25		2.13	1.50			
		explained variance (cummul.)	0.44	0.73		0.40	0.76		0.45	0.77		0.43	0.73		NA	
		variables	variable importance		variables	variable importance		variables	variable importance		variables	variable importance		variables	variable importance	
	DCMU	F440	0.09	- 0.84	F690	0.43	- 0.52	F440	0.43	0.20	F440	0.37	0.50			
		F690	- 0.62	- 0.15	F440/F520	0.39	0.47	F690	0.27	- 0.53	F740	0.48	- 0.34			
		F440/F520	0.52	0.37	F520/F740	- 0.67	0.05	F440/F520	- 0.48	0.21	F520/F690	- 0.20	0.77		NA	
		F690/F740	- 0.58	0.36	F690/F740	- 0.44	- 0.34	F440/F690	0.06	0.66	F690/F740	0.51	0.04			
					Fv/Fm	- 0.14	0.62	F520/F740	0.41	0.43	Fv/Fm	- 0.58	- 0.20			
								F690/F740	0.35	- 0.11						
								Fv/Fm	- 0.47	0.06						
			PC1	PC2		PC1	PC2		PC1	PC2		PC1	PC2		PC1	PC2
		eigenvalue	2.38	1.95		1.85	1.36		2.26	1.34		3.67	0.99		2.62	2.35
		explained variance (cummul.)	0.40	0.72		0.46	0.80		0.56	0.90		0.61	0.78		0.44	0.83
		variables	variable importance		variables	variable importance		variables	variable importance		variables	variable importance		variables	variable importance	
	glyphosate	F440	0.14	- 0.64	F690	0.61	- 0.31	F690	0.59	- 0.33	F440	0.31	0.61	F440	- 0.12	- 0.56
		F690	0.54	- 0.27	F520/F740	- 0.65	- 0.11	F520/F740	- 0.34	0.73	F690	0.50	- 0.13	F740	- 0.58	0.04
		F440/F520	0.00	0.40	F690/F740	- 0.42	- 0.58	F690/F740	0.49	0.49	F440/F520	- 0.37	0.03	F440/F520	0.40	0.39
		F440/F740	- 0.60	- 0.18	Fv/Fm	- 0.17	0.75	Fv/Fm	- 0.54	- 0.36	F440/F740	- 0.31	0.75	F520/F690	0.54	- 0.23
		F520/F690	- 0.43	- 0.52							F690/F740	0.44	0.17	F520/F740	0.37	- 0.52
		F690/F740	- 0.39	0.25							Fv/Fm	- 0.48	- 0.10	F690/F740	- 0.26	- 0.46
			PC1	PC2		PC1	PC2		PC1	PC2		PC1	PC2		PC1	PC2
		eigenvalue	1.58	1.22		1.81	1.40		1.59	1.46		2.40	0.93		2.35	0.41
		explained variance (cummul.)	0.40	0.70		0.45	0.80		0.40	0.76		0.60	0.83		0.78	0.92
		variables	variable importance		variables	variable importance		variables	variable importance		variables	variable importance		variables	variable importance	
	chromium	F690	- 0.49	0.48	F440	0.14	0.65	F690	0.52	- 0.47	F690	0.58	- 0.30	F440	0.56	0.77
		F440/F520	0.36	- 0.65	F740	- 0.63	0.45	F440/F520	- 0.59	0.36	F440/F520	- 0.48	0.62	F740	0.60	- 0.11
		F690/F740	0.57	0.37	F520/F690	0.74	0.08	F690/F740	- 0.52	- 0.48	F690/F740	0.44	0.59	Fv/Fm	- 0.57	0.63
		Fv/Fm	- 0.56	- 0.46	F690/F740	- 0.21	- 0.61	Fv/Fm	0.33	0.64	Fv/Fm	- 0.49	- 0.43			
			PC1	PC2		PC1	PC2		PC1	PC2		PC1	PC2		PC1	PC2
		eigenvalue	1.66	1.22		2.20	1.54		1.94	1.30		2.24	0.76			
		explained variance (cummul.)	0.41	0.72		0.37	0.62		0.48	0.81		0.56	0.75		NA	
		variables	variable importance		variables	variable importance		variables	variable importance		variables	variable importance		variables	variable importance	
	pooled data for all toxicants	F440	0.19	- 0.78	F440	0.18	- 0.39	F440	0.55	- 0.31	F440	0.50	- 0.24			
		F440/F520	0.44	0.51	F690	0.64	- 0.02	F440/F520	- 0.56	0.39	F740	0.53	- 0.04			
		F690/F740	- 0.62	0.31	F440/F520	- 0.18	0.60	F690/F740	0.22	0.79	F440/F520	- 0.54	0.41		NA	
		Fv/Fm	0.62	0.19	F520/F740	- 0.52	- 0.47	Fv/Fm	- 0.59	- 0.36	F690/F740	0.42	0.88			
					F690/F740	- 0.12	- 0.47									
					Fv/Fm	- 0.49	0.22									

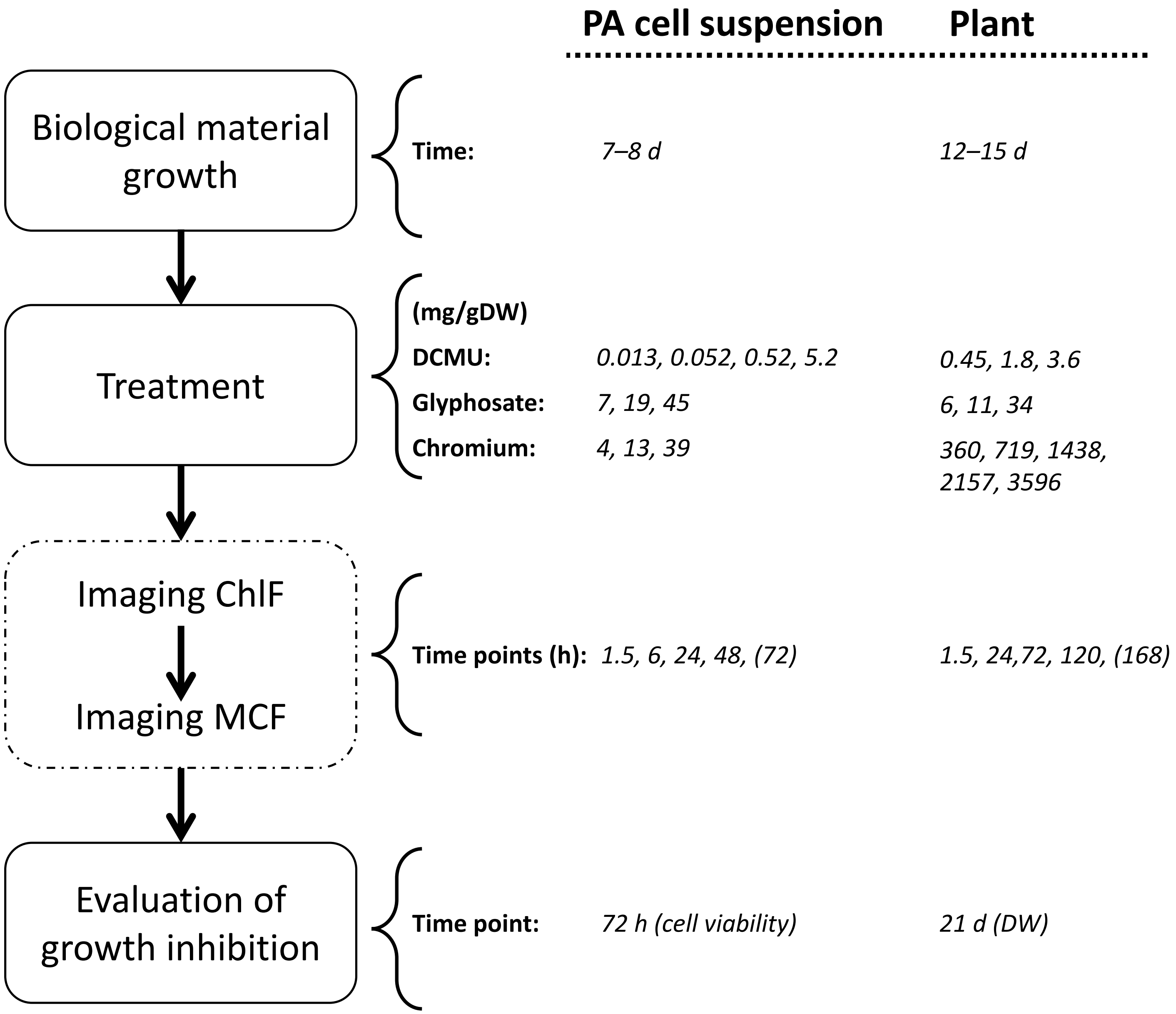

Fig. S19 Flowchart of experimental design.

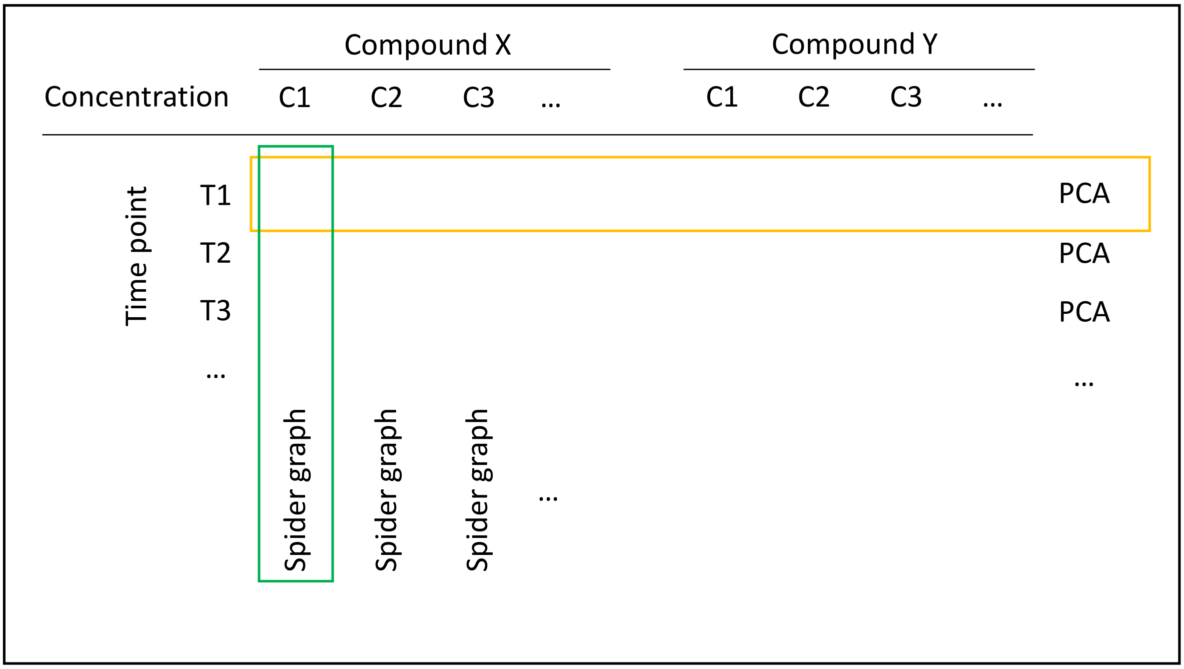

Fig. S20 Scheme of data organization and inputs used for analyses.
